# Thiazole formation through a modified Gewald reaction

**DOI:** 10.3762/bjoc.11.98

**Published:** 2015-05-26

**Authors:** Carl J Mallia, Lukas Englert, Gary C Walter, Ian R Baxendale

**Affiliations:** 1 Department of Chemistry, Durham University, South Road, Durham, DH1 3LE, United Kingdom; 2 Syngenta CP R&D Chemistry, Jealott's Hill International Research Centre, Bracknell, Berkshire, RG42 6EY, United Kingdom

**Keywords:** design of experiment (DOE), 1,4-dithiane-2,5-diol, Gewald reaction, thiazole, thiophene

## Abstract

The synthesis of thiazoles and thiophenes starting from nitriles, via a modified Gewald reaction has been studied for a number of different substrates. 1,4-Dithiane-2,5-diol was used as the aldehyde precursor to give either 2-substituted thiazoles or 2-substituted aminothiophenes depending on the substitution of the α-carbon to the cyano group.

## Introduction

**Figure 1:**
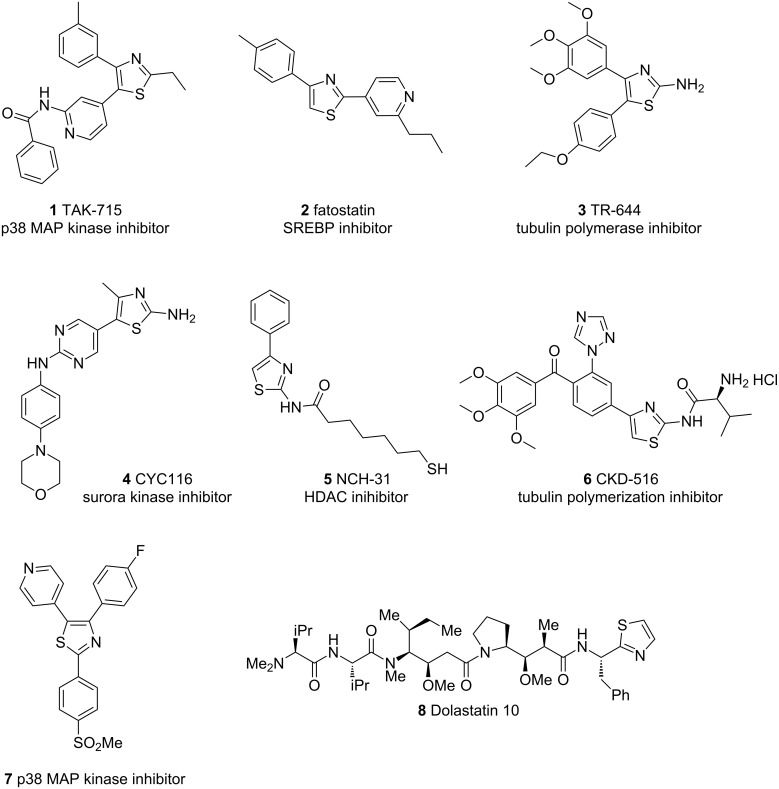
Selected examples of biologically active thiazole containing molecules [12–20].

Thiazoles are privileged motifs which are encountered in many naturally occurring bioactive compounds and pharmaceuticals with indications in a number of therapeutic areas including anticancer, antifungal, antibacterial, anti-inflammatory and as antidepressants (Figure 1) [1]. Several protocols have already been described for the synthesis of substituted thiazoles and benzothiazoles [2–11]. The current first choice synthesis protocol, the Hantzsch synthesis, requires access to appropriately functionalised α-halocarbonyls but such starting materials are not always readily available. Consequently, expanding the scope of thiazole synthesis by developing new methodologies remains an active area of research.

The air-stable, readily available 1,4-dithian-2,5-diol (**10**) has been previously reported as a coupling partner with various nitriles to give 2-aminothiophenes through a Gewald mechanism [21–22]. However, we have noticed that α-substituted benzylacetonitriles, alternatively yield 2-substituted thiazoles, when coupled with the aldehyde derived from **10**. Although the thiazole formation from nitriles has already been shown to occur with ketones [23–26] and carboxylic acid [27–28] derivatives to give 2,5-disubstituted thiazoles, to our knowledge aldehydes have only been shown to form 2-aminothiophenes [29]. Even though 2-substituted thiazoles are important structures in their own right, further substitution can be easily achieved through published protocols to form 2,4-substituted thiazoles, 2,5-substituted thiazoles and also 2,4,5-substituted thiazoles [30]. This further shows the need for the rapid and facile formation of 2-substituted thiazole compounds as core building blocks.

## Results and Discussion

### Screening for the bifurcation conditions

**Scheme 1:**
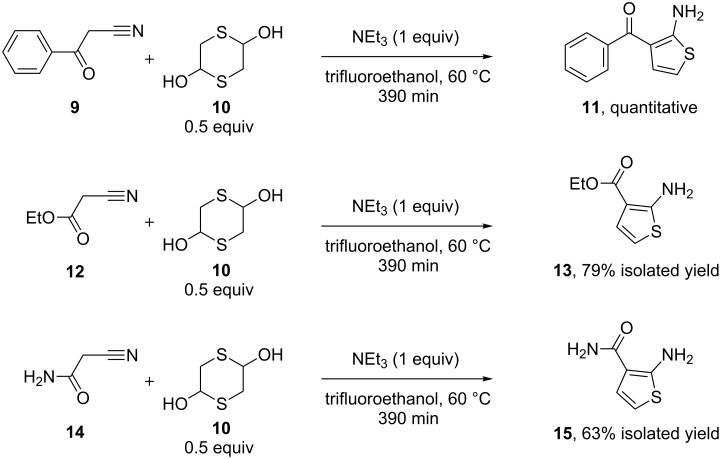
Illustration of substrates that form thiophenes under Gewald-type conditions.

Initially, the reaction of several nitriles was screened with aldehyde precursor **10** to determine the selectivity between thiophene and thiazole products. In general, substrates which have an α-methylene adjacent to the nitrile group gave 2-aminothiophene (Scheme 1) whilst those that possessed an α-methine yielded the corresponding 2-substituted thiazole. Originally, reactions were performed using conventional heating, however, to allow for a wider temperature range, microwave heating was introduced. This allowed access to higher temperatures above the boiling points of the solvents used at atmospheric pressure.

### Optimisation and scoping of thiazole formation

The reaction of ethyl phenylcyanoacetate (**16**) with pro-aldehyde **10** was used as a model system applying a fixed temperature of 80 °C and a reaction time of 300 min to evaluate different solvents, bases and stoichiometry of **10** (Table 1). Only a small impact upon conversion was observed with changing the ratio of compound **10** and so it was decided to also maintain this at equimolar concentration (i.e., 0.5 equiv of the dimer) as it would simplify the purification later on. All reactions were assessed for conversion using ^1^H NMR analysis of a crude sample followed by work-up and purification via column chromatography to determine the yields.

**Table 1:** Scoping experiments using ethyl phenylcyanoacetate (**16**) with different solvents and bases.^a^

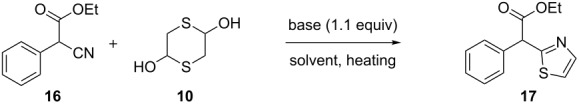					

Entry	Base	Solvent	Ditiane (equiv)	Conversion^b^ (%)	Isolated yield

1	NEt_3_	trifluoroethanol	0.50	84	58
2	NEt_3_	trifluoroethanol	0.55	85	50
3	NEt_3_	trifluoroethanol	0.75	94	52
4	NEt_3_	ethanol	0.50	25	N/D
5	NEt_3_	chlorobenzene	0.50	18	N/D
6	NEt_3_	1,2-dichloroethane	0.50	0	N/D
7	DBU	trifluoroethanol	0.50	100	5
8	TMG	trifluoroethanol	0.50	100	33
9	piperidine	trifluoroethanol	0.50	0	0
10	TMEDA	trifluoroethanol	0.50	67	50
11	QP-SA	trifluoroethanol	0.50	0	0

^a^The reactions were carried out at 80 °C for 300 min and at a concentration of 0.143 M. ^b^Conversion of starting material to product was measured by ^1^H NMR spectroscopy. N/D: not determined.

With regard to solvent selection, trifluroethanol showed by far the best results, this is probably due to the high polarity and its slightly acidic nature, which assists in solubilising **10**, and subsequently promotes the formation of the aldehyde monomer. The base screen indicated that triethylamine (NEt_3_) was the most effective base providing the highest conversion and isolated yield (58%) with tetramethylethylenediamine (TMEDA) also giving a respectable yield of 50% (Table 1, entries 1 and 10). The stronger guanidine bases 1,1,3,3-tetramethylguanidine (TMG) and 1,8-diazabicycloundec-7-ene (DBU) both gave full consumption of the nitrile starting material, but generated complicated product mixtures allowing only a moderate isolated yield of 33% for the TMG and negligible recovery for the DBU (Table 1, entries 7 and 8). Interestingly, the use of piperidine led to no conversion under these reaction conditions (Table 1, entry 9), we believe this is due to its condensation with the aldehyde component (generated from **10**), which inhibits the transformation. In addition, a sulfonic acid bound resin (QP-SA) was also trialled as an additive but showed no conversion allowing full recovery of the starting nitrile (see later discussion on mechanism). These experiments imply that the deprotonation of the α-methylene adjacent to the nitrile group is an essential part of the mechanism.

In an attempt to improve the yield of this reaction, a design of experiment analysis (DOE) was performed initially testing three factors; temperature, concentration of **16** and reaction time, while monitoring the response by measuring the isolated yield of **17**. The starting point for the design of the array was the best conditions obtained from the initial scoping (Table 1, entry 1), this generated the profiles and results as shown in Table 2 and Table 3. From the data it was concluded that elevated temperatures resulted in lower isolated yields most probably due to decomposition of compound **10** or the resulting aldehyde. In general, lower concentration was beneficial but at a consequence of longer reaction times in order to obtain a good yield. The best results were entry 9, Table 2 and entries 8 and 9, Table 3 which produced similar results. The latter conditions were then chosen to progress due to the increased productivity based upon the higher concentration and slightly shorter reaction time.

**Table 2:** 1^st^ Full factorial screening for conversion of compound **16**.

Entry	Pattern^a^	Temperature (°C)	Time (min)	Concentration of **16** (M)	Isolated yield (%)

1	−++	40	420	0.21	70
2	++−	120	420	0.07	31
3	000	80	300	0.14	51
4	+++	120	420	0.21	18
5	+−−	120	180	0.07	43
6	+−+	120	180	0.21	33
7	−−+	40	180	0.21	67
8	−−−	40	180	0.07	68
9	−+−	40	420	0.07	85
10	000	80	300	0.14	58

^a^Where ‘+’ refers to the maximum limit, ‘−’ refers to the minimum limit and ‘000’ refers to the middle limits.

**Table 3:** 2^nd^ Factorial screening for **16**.

Entry	Pattern^a^	Temperature (°C)	Time (min)	Concentration of **16** (M)	Isolated yield (%)

1	++−	80	480	0.04	71
2	−−−	40	300	0.04	66
3	+++	80	480	0.18	36
4	+−+	80	300	0.18	52
5	−+−	40	480	0.04	76
6	−++	40	480	0.18	56
7	+−−	80	300	0.04	66
8	000	60	390	0.11	83
9	000	60	390	0.11	81
10	−−+	40	300	0.18	76

^a^Where ‘+’ refers to the maximum limit, ‘−’ refers to the minimum limit and ‘000’ refers to the middle limits.

**Figure 2:**
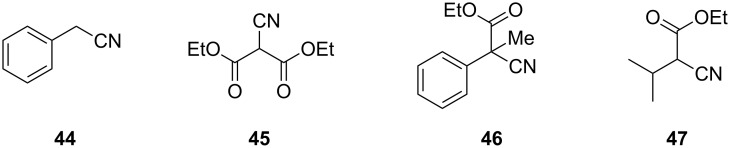
Substrates which did not react under the optimised conditions.

Having established an optimised set of conditions for the formation of thiazole **17**, we next turned our attention to expanding the versatility of the reaction by changing both the aromatic portion and the ester functionality of the substrate (Table 4). To allow direct comparison and evaluation of the influence of substrate modifications on the reaction outcome we maintained the standard reaction conditions generated above. It should be noted that these reactions are therefore not optimised and that improvement could be achieved as highlighted by entries 14 and 15 in Table 4. In general esters, amides and nitriles are tolerated, with methyl esters giving generally lower yields (hydrolysis occurs from generated water) than the corresponding ethyl analogues (entries 1, 2 and 9, 10, Table 4). The isopropyl ester leads to a lower conversion and isolated yield presumably as a consequence of steric interactions (entry 3, Table 4). Changing the electronic character of the aromatic appendage shows that an electron donating group (entries 4 and 5, Table 4) gives a better yield than an electron withdrawing group (entries 6 and 7, Table 4). This is presumably due to a subtle balance between the basicity and nucleophilicity of the intermediate anion, which could in the case of the more stable (electron withdrawing group) anion enable a retro-aldol reaction following. Substituted malonitriles are also tolerated forming the corresponding cyanothiazole in good yield, with no indication of the dithiazole product observed (entry 8, Table 4). Benzyl groups are also tolerated (entries 9–12, Table 4) but give lower yields than the corresponding aromatics. Changing to an aliphatic group instead of the aromatic moiety (entries 9–15, Table 4) decreases the conversion, sterics again seem to play an important role. Substrate possessing a methyl or an ethyl group react well (entries 13 and 14, Table 4) but an isopropyl group such as in molecule **47** (Figure 2), reproducibly gave no product, indicating the steric limits of the reaction.

**Table 4:** Scoping of the 2-substituted thiazole formation.^a^

Entry	Starting material	Product	Isolated yield (%)

1	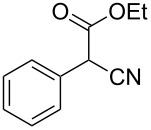 **16**	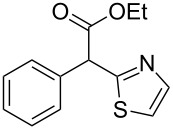 **17**	83
2	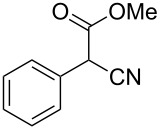 **18**	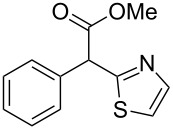 **19**	60
3	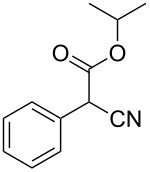 **20**	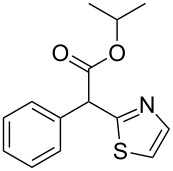 **21**	49
4	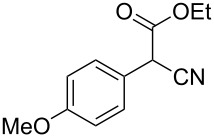 **22**	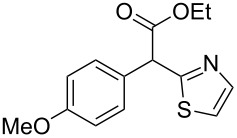 **23**	57
5	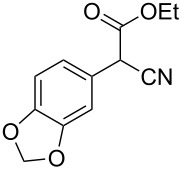 **24**	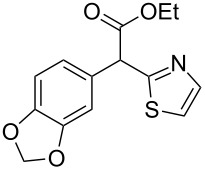 **25**	51
6	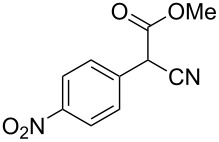 **26**	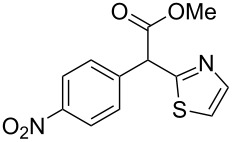 **27**	36
7	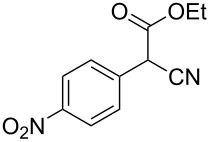 **28**	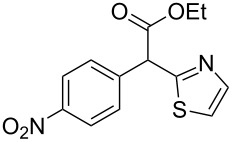 **29**	35
8	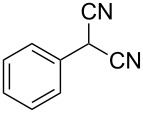 **30**	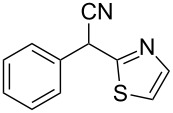 **31**	73
9	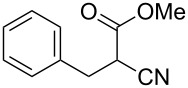 **32**	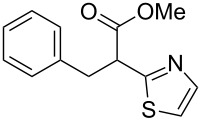 **33**	16
10	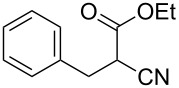 **34**	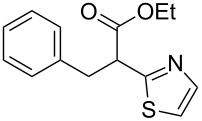 **35**	36
11	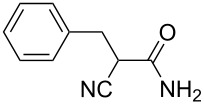 **36**	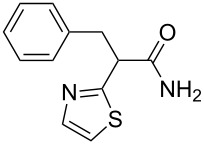 **37**	34
12	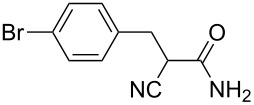 **38**	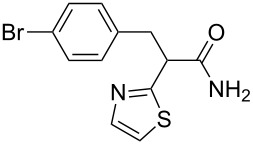 **39**	37
13	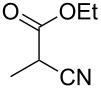 **40**	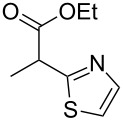 **41**	35
14	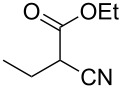 **42**	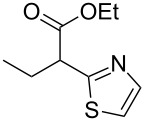 **43**	33
15^b^	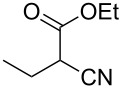 **42**	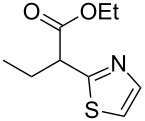 **43**	45

^a^Conditions: 0.50 equiv 1,4-dithian-2,5-diol (**10**), 0.11 M of nitrile, 1.10 equiv NEt_3_, 2 mL trifluoroethanol, 60 °C, 390 min; ^b^0.50 equiv 1,4-dithian-2,5-diol (**10**) followed by another 0.50 equiv of **10** after the first 390 min, 0.11 M of nitrile, 1.10 equiv NEt_3_, 2 mL trifluoroethanol, 80 °C, 630 min.

Although the reaction appears general, certain substrates tested did not generate any product when employing the standard reaction conditions (Figure 2) thus helping to identify certain attributes of the mechanism. For example, 2-phenylacetonitrile (**44**) failed to react most likely due to the lower acidity of the α-methylene protons. Diethyl 2-cyanomalonate (**45**), also proved unreactive, this substrate would be expected to form an extensively delocalised anion which would be a poor nucleophile. Also, substrate **46**, lacking an acidic proton, was recovered unchanged from the reaction. Finally, compound **47**, as mentioned previously, possesses a high degree of steric hindrance around the α-carbon to the cyano group thus inhibiting the reaction.

**Scheme 2:**
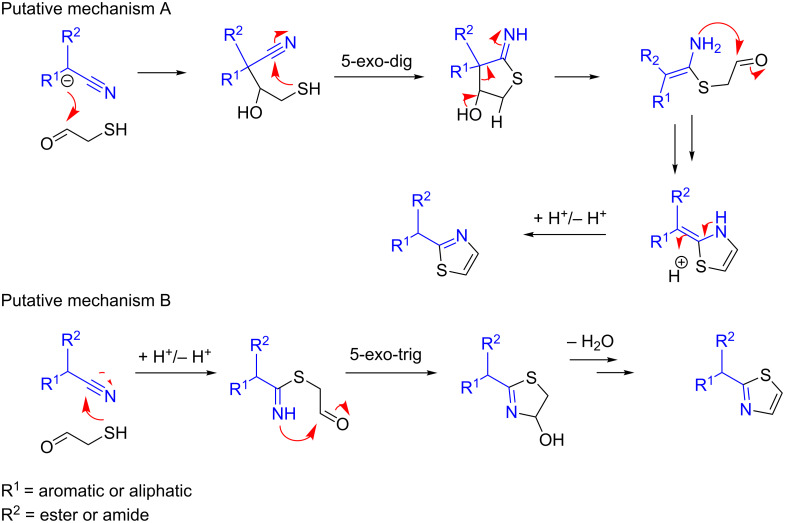
Proposed mechanisms for the formation of thiazoles.

The original pathway for the thiophene formation (Scheme 1) follows the Gewald mechanism and has already been described [31]. However, the alternative mechanism for the thiazole formation as described herein has not previously been reported and initially presented some queries. We envisaged two putative mechanisms for the formation of the thiazole (Scheme 2), which could both be involved in the formation depending on the specific substrate involved. Putative mechanism A is theoretically valid when a methine or methylene group is present in the α-position to the nitrile group, which is reminiscent of the original Gewald reaction mechanism. Mechanism B would also be viable for molecules which possess no protons α to the nitrile group. The fact that compounds **46** and **47** did not react implies mechanism A is the predominant pathway. The lack of reactivity of substrate **47** can be attributed to the high degree of steric hindrance inhibiting its enolisation. In summary, although our evidence indicates mechanism A is the most likely pathway it should be noted that several benzonitrile derivatives have been shown to successfully result in thiazole formation when reacted with coupling partners such as 2-mercaptopropionic acid, therefore mechanism B could operate under certain conditions [32].

## Conclusion

We have successfully shown that substitution of the nitrile precursor predetermines the reaction outcome yielding exclusively to a thiophene or thiazole product. It was shown that the presence of an alkyl or aryl substituent adjacent to the cyano group leads selectively to the thiazole by blocking the Gewald type mechanism responsible for the formation of the 2-aminothiophene. In the study, the thiazole formation from the appropriately substituted α-methine nitrile compounds was evaluated, demonstrating its scope as an effecient way of synthesising 2-subsituted thiazoles from readily available, air stable 1,4-dithiane-2,5-diol (**10**) as a precursor for 2-mercaptoacetaldehyde.

## Experimental

See Supporting Information File 1 for full experimental data.

## Supporting Information

File 1 Experimental and analytical data.
