# Supplementary material for: Thiazole formation through a modified Gewald reaction
Source: Beilstein J Org Chem. 2015 May 26;11:875–83. doi: 10.3762/bjoc.11.98 (PMC4464301; doi:10.3762/bjoc.11.98)
Supplement: File 1 — Experimental and analytical data. [file Beilstein_J_Org_Chem-11-875-s001.pdf]

# Supporting Information

# for

# Thiazole formation through a modified Gewald reaction

Carl J. Mallia[1], Lukas Englert[1], Gary C. Walter[2] and Ian R. Baxendale[*1§]

Address: [1]Department of Chemistry, Durham University, South Road, Durham, DH1 3LE, United Kingdom and [2]Syngenta CP R&D Chemistry, Jealott's Hill International Research Centre, Bracknell, Berkshire, RG42 6EY, United Kingdom

Email: Ian R. Baxendale - i.r.baxendale@durham.ac.uk

[§]Tel: +44 191 334 2185

*Corresponding author

## Experimental and analytical data

## Table of Contents

**Materials and methods ............................................................................................S2**

**General procedure for thiazole and thiophene synthesis ..........................................S3**

**Spectroscopic data for thiophenes and thiazoles.......................................................S3**

**Synthesis and spectroscopic data of starting materials .............................................S9**

## Materials and methods

Unless specified, reagents were obtained from commercial sources and used without further purification. Solvents were obtained from Fisher scientific, and $H_2O$ was deionised before use. Analytical grade THF was dried using Innovative Technology Inc. solvent purification system and stored under nitrogen.

NMR spectra were recorded on either Bruker Avance-400, Varian VNMRS-600 or Varian VNMRS-700 instruments and are calibrated to the residual solvent according to the literature.[1] Assignments are based on DEPT-135, COSY, HSQC and HMBC spectra.

Liquid chromatography-mass spectrometry (LCMS) was performed on an Agilent HP 1100 series chromatograph (Mercury Luna 3µ C18 (2) column) attached to a Waters ZQ2000 mass spectrometer with ESCi ionisation source in ESI mode. Elution was carried out at a flow rate of 0.6 mL/min using a reverse phase gradient of MeCN–water containing 0.1% formic acid. Gradient = 0–1 min: hold MeCN 5%, 1–4 min: ramp MeCN 5–95%, 4–5 min: hold MeCN 95%, 5–7 min: ramp MeCN 95–5%, 7–8 min: hold MeCN 5%. Retention times are reported as Rt.

High resolution mass spectra (HRMS) were recorded on a Waters Micromass LCT Premier spectrometer using time of flight with positive electrospray ionisation (ESI+), an ABI/MDS Sciex Q-STAR Pulsar with ESI+ and an ASAP (atmospheric pressure solids analysis probe ionisation), or a Bruker BioApex II 4.7e FTICR utilising either ESI+ or a positive electron ionisation (EI+) source equipped with a direct insertion probe. The mass reported is that containing the most abundant isotopes ($^{35}$Cl and $^{79}$Br). Limit: ± 5 ppm.

IR spectra were recorded neat on a Perkin-Elmer Spectrum One or Perkin-Elmer Spectrum Two FTIR spectrometer using Universal ATR sampling accessories. Letters in parentheses refer to the relative absorbency of the peak: w – weak (<40% of the most intense peak), m – medium (40–75% of the most intense peak), s – strong (>75% of the most intense peak) and br – broad.

Melting points were recorded on an Optimelt automated melting point system with a heating rate of 1 °C/min (70% onset point and 10% clear point) and are uncorrected.

Microwave heating was performed using a Biotage® Initiator or Initiator$^+$.

[1] Gottlieb, H. E.; Kotlyar, V.; Nudelman, A. *J. Org. Chem.* **1997**, *62*, 7512-7515.

## General procedure for thiazole and thiophene synthesis

In a 2–5 mL microwave vial was added the nitrile (0.22 mmol, 1 equiv) and trifluoroethanol (2 mL) which was stirring for 2 min to dissolve. Next, 1,4-dithian-2,5-diol (0.11 mmol, 0.5 equiv) was then added and stirred for 5 min before triethylamine (0.242 mmol; 1.1 equiv) was added and the mixture further stirred for 2 min. The vial was then sealed and heated in the microwave for 390 min at 60 °C. The solvent was evaporated under *vacuo* and the crude residue was then purified using flash chromatography on silica (EtOAc/hexanes).

## Spectroscopic data for thiophenes and thiazoles:

**(2-Aminothiophen-3-yl)(phenyl)methanone (11):**

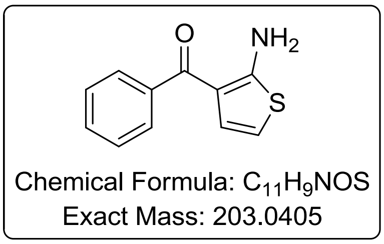


Isolated yield: 45 mg (quantitative, 0.22 mmol scale), yellow crystalline product; $^{1}$H NMR (400 MHz, $CDCl_3$) δ/ppm 6.16 (d, *J* = 6.0 Hz, 1H), 6.91 (d, *J* = 6.0 Hz, 1H), 7.42-7.57 (m, 3H), 7.70 (d, *J* = 7.2 Hz, 2H). $^{13}$C NMR (101 MHz, $CDCl_3$) δ/ppm 106.1, 115.1, 127.8, 128.1, 128.1, 130.7, 140.8, 166.1, 191.2; IR (neat) ν = 3359 (m), 3241 (br), 3128 (w), 1600 (m), 1570 (m), 1444 (s), 1420 (s), 1418 (s), 1398 (m), 1325 (m), 1289 (m), 1213 (w), 1078 (w), 946 (w), 833 (s), 698 (s), 672 (s), 664 (m), 654 (m), 587 (m), 494 (s) cm$^{-1;}$ HR-MS ($^{+}$ESI-TOF) calculated 204.0483, found 204.0475 (Δ = 3.9 ppm); Melting point: 151-153 °C.

**Ethyl 2-aminothiophene-3-carboxylate (13):**

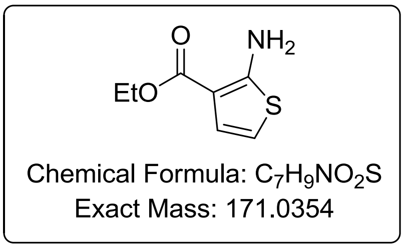


Isolated yield: 30 mg (79%, 0.22 mmol scale), colourless oil; $^{1}$H NMR (400 MHz, $CDCl_3$) δ/ppm 7.00 (d, *J* = 5.6 Hz, 1H), 6.20 (d, *J* = 5.6 Hz, 1H), 5.93 (s, 2H), 4.30 (dd, *J* = 7.0, 14.2 Hz, 2H),1.36 (t, *J* = 7.0 Hz, 3H); $^{13}$C NMR (101 MHz, $CDCl_3$) δ/ppm 165.4, 162.6, 125.9, 107.3, 106.9, 59.7, 14.5; IR (neat) ν = 3435 (w), 3326 (w), 1650 (m), 1582 (m), 1522 (m), 1485 (m), 1400 (m), 1380 (m), 1305 (m), 1261 (m), 1169 (w), 1108 (m), 1070 (w), 1020 (m), 901 (w), 828 (w), 783 (w), 679 (m) cm$^{-1}$; HR-MS ($^{+}$ESI-TOF) calculated 172.0423, found 172.0424 (Δ = 0.6 ppm).

**2-Aminothiophene-3-carboxamide (15):**

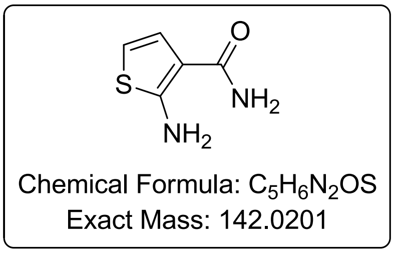


Isolated yield: 37 mg (63%, 0.44 mmol scale), pink, amorphous solid; $^{1}$H NMR (400 MHz, DMSO-$d_6$) δ/ppm 7.46–7.10 (m, 3H), 7.04 (d, *J* = 5.8 Hz, 1H), 6.80 (d, *J* = 50.6 Hz, 1H), 6.22 (d, *J* = 5.8 Hz, 1H); $^{13}$C NMR (101 MHz, $CDCl_3$) δ/ppm 168.0, 162.1, 125.3, 107.6, 105.8; IR (neat) ν = 3438 (br), 3409 (br), 3321 (br), 3261 (br), 3208 (br), 3119 (w), 3083 (w), 1645 (m), 1594 (s), 1562 (s), 1559 (s), 1520 (s), 1417 (m), 1418 (s), 1348 (m), 1284 (w), 1220 (w), 1101 (m), 895 (w), 872 (w), 785 (m), 690 (m), 664 (m) cm$^{-1}$. LC-MS (MeOH), Rt. 1.47 min, m/z = 142.9 $[M+H]^{+}$. HR-MS ($^{+}$ESI-TOF) calculated for $C_{10}H_9N_2O$ 142.0279, found 142.0278 (Δ = 0.7 ppm); Melting point: 130 °C (decomposed, EtOH).

**Ethyl 2-phenyl-2-(thiazol-2-yl)acetate (17):**

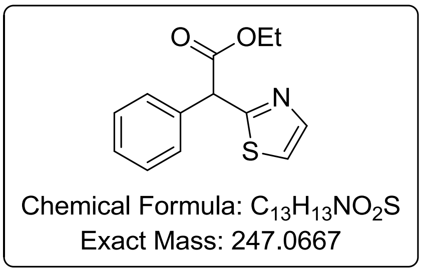


Isolated yield: 45 mg (83%, 0.22 mmol scale), pale yellow oil; Rf: 0.37 (1:4, EtOAc/hexane); $^{1}$H NMR (700 MHz, $CDCl_3$): δ/ppm 7.75 (d, *J* = 3.3 Hz, 1H), 7.45 (d, *J* = 7.4 Hz, 2H), 7.39–7.35 (m, 2H), 7.34–7.30 (m, 1H), 7.29 (d, *J* = 3.3 Hz, 1H), 5.42 (s, 1H), 4.30–4.19 (m, 2H), 1.26 (t, *J* = 7.1 Hz, 3H); $^{13}$C NMR (176 MHz, $CDCl_3$) δ/ppm 170.6 (C=O), 167.6, 142.5, 136.7, 129.1, 128.6, 128.3, 120.1, 77.3, 77.2, 76.9, 62.0, 55.6, 14.2; IR (neat) ν = 3064 (s), 3031 (s), 2980 (s), 2935 (s), 1730 (w), 1644 (s), 1600 (s), 1494 (m), 1454 (m), 1421 (s), 1390 (s), 1367 (m), 1308 (m), 1231 (m), 1185 (w), 1153 (w), 1130 (m), 1094 (m), 1056 (m), 1021 (w), 916 (s), 863 (m), 789 (m), 724 (w), 698 (w), 644 (s), 610 (m) $cm^{-1}$; LC-MS (MeCN), Rt. 2.79 min, m/z = 248.8 $[M+H]^+$. HR-MS ($^+$ESI-TOF) calculated for $C_{13}H_{14}NO_2S$ 248.0745, found 248.0755 (Δ = 4.0 ppm).

**Methyl 2- phenyl-2-(thiazol-2-yl)acetate (19):**

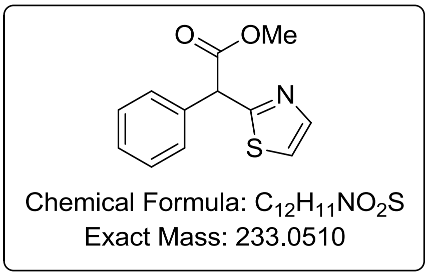


Isolated yield: 40 mg (60%, 0.22 mmol scale), pale yellow oil; Rf: 0.1 (1/9, EtOAc/hexane); $^{1}$H NMR (700 MHz, $CDCl_3$) δ/ppm 7.79–7.75 (m, 1H), 7.46 (d, *J* = 8.3 Hz, 2H), 7.40–7.32 (m, 3H), 7.32–7.29 (m, 1H), 5.46 (s, 1H), 3.79 (d, *J* = 1.5 Hz, 3H); $^{13}$C NMR (176 MHz, $CDCl_3$) δ/ppm 170.9, 167.5, 142.7, 142.2, 136.4, 128.9, 128.7, 128.5, 128.3, 128.2, 126.5, 120.6, 120.1, 77.2, 76.9, 76.8, 55.2, 52.9; IR (neat) ν = 3087.8 (w), 2952.3 (w), 1967.9 (w), 1732.2 (s), 1644.1 (w), 1599.0 (w), 1494.2 (w), 1448.3 (w), 1434.2 (w), 1387.9 (w), 1368.0 (w), 1312.9 (w), 1240.8 (m), 1198.9 (m), 1156.3 (m), 1132.9 (m), 1098.1 (w), 1058.2 (w), 1005.4 (m), 892.0 (w), 863.0 (w), 794.8 (w), 728.3 (s), 696.6 (s), 653.5 (w), 612.1 (m) $cm^{-1}$; LC-MS (MeCN), Rt. 2.63 min, m/z = 233.9 $[M+H]^+$. HR-MS ($^+$ESI-TOF) calculated for $C_{12}H_{12}NO_2S$ 234.0589, found 234.0592 (Δ = 1.3 ppm).

**Isopropyl 2-phenyl-2-(thiazol-2-yl)acetate (21):**

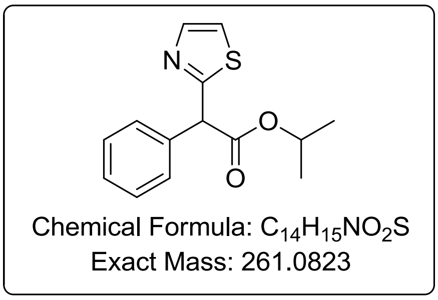


Isolated yield: 28 mg (49%, 0.22 mmol scale), colourless oil; $^{1}$H NMR (400 MHz, $CDCl_3$) δ/ppm 7.75 (d, *J* = 3.3 Hz, 1H), 7.47–7.30 (m, 5H), 7.29 (d, *J* = 3.3 Hz, 1H), 5.38 (s, 1H), 5.11 (hept, *J* = 6.3 Hz, 1H), 1.27 (d, *J* = 6.3 Hz, 3H), 1.19 (d, *J* = 6.2 Hz, 3H); $^{13}$C NMR (101 MHz, $CDCl_3$) δ/ppm 170.0, 167.6, 142.4, 136.8, 128.9, 128.4, 128.1, 120.0, 69.6, 55.7, 21.7, 21.5; IR (neat) ν = 3111 (w), 3088 (w), 3063 (w), 2982 (w), 2936 (w), 1727 (s), 1646 (w), 1599 (w), 1495 (w), 1449 (w), 1387 (w), 1243 (m), 1168 (m), 1100 (s), 1068 (m), 1003 (w), 863 (w), 729 (m), 714 (m), 697 (s); LC-MS (MeCN), Rt. 3.07 min, m/z = 262.1 $[M+H]^+$. HR-MS ($^+$ESI-TOF) calculated for $C_{14}H_{16}NO_2S$ 262.0902, found 262.0900 (Δ = 0.8 ppm).

**Ethyl 2-(4-methoxyphenyl)-2-(thiazol-2-yl)acetate (23):**

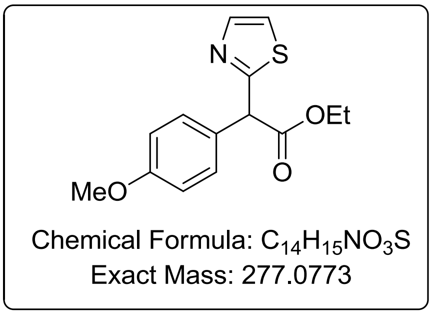


Isolated yield: 69 mg (57%, 0.44 mmol scale), brown oil; $R_f$: 0.17 (2/8, EtOAc/hexane); $^1$H NMR (700 MHz, $CDCl_3$) δ/ppm 7.76 (d, *J* = 3.3 Hz, 1H), 7.37–7.40 (m, 2H), 7.30 (d, *J* = 3.3 Hz, 1H), 6.87–6.91 (m, 2H), 5.39 (s, 1H), 4.19–4.29 (m, 2H), 3.80 (s, 3H), 1.26 (t, *J* = 7.1 Hz, 3H); $^{13}$C NMR (176 MHz, $CDCl_3$) δ/ppm 170.8, 168.5, 159.6, 142.1, 129.8, 128.7, 120.2, 114.5, 62.1, 55.4, 54.6, 14.2; IR (neat) ν = 3849 (w), 3735 (w), 3648 (w), 2982 (w), 2934 (w), 2900 (w), 2836 (w), 2168 (w), 2154 (w), 2130 (w), 2047 (w), 2040 (w), 2024 (w), 1730 (s), 1608 (m), 1509 (s), 1462 (m), 1442 (m), 1421 (w), 1391 (w), 1367 (w), 1302 (m), 1249 (s), 1177 (s), 1156 (s), 1129 (m), 1093 (m), 1055 (w), 1023 (s), 867 (w), 835 (m), 796 (m), 777 (w), 728 (m), 609 (w) $cm^{-1}$; LC-MS (MeCN), Rt. 2.77 min, m/z = 278.5 $[M+H]^+$. HR-MS ($^+$ESI-TOF) calculated for $C_{14}H_{16}NO_3S$ 278.0851, found 278.00853 (Δ = 0.7 ppm).

**Ethyl 2-(benzo[*d*][1,3]dioxol-5-yl)-2-(thiazol-2-yl)acetate (25):**

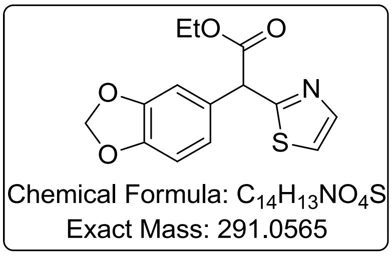


Isolated yield: 65 mg (51%, 0.44 mmol scale), yellow oil; $^1$H NMR (400 MHz, $CDCl_3$) δ/ppm 7.75 (d, *J* = 3.3 Hz, 1H), 7.29 (d, *J* = 3.3 Hz, 1H), 6.96 (d, *J* = 1.8 Hz, 1H), 6.90 (ddd, *J* = 8.0, 1.8, 0.5 Hz, 1H), 6.78 (d, *J* = 8.0 Hz, 1H), 5.95 (s, 2H), 5.30 (s, 1H), 4.31–4.16 (m, 2H), 1.26 (t, *J* = 7.1 Hz, 3H); $^{13}$C NMR (101 MHz, $CDCl_3$) δ/ppm 170.5, 167.7, 148.0, 147.6, 142.5, 130.2, 122.1, 120.0, 109.00, 108.5, 101.3, 62.0, 55.0, 14.01; IR (neat) ν = 3125 (w), 3082 (w), 2986 (w), 2902 (w), 1732 (s), 1503 (m), 1488 9s), 1444 (m), 1311 (w), 1244 (s), 1186 (s), 1162 (s), 1130 (w), 1099 (w), 1036 (s), 929 (m), 813 (w), 772 (w), 731 (w) $cm^{-1}$; LC-MS (MeCN), Rt. 2.67 min, m/z = 292.0 $[M+H]^+$. HR-MS ($^+$ESI-TOF) calculated for $C_{14}H_{14}NO_4S$ 292.0644, found 292.0641 (Δ = 1.0 ppm).

**Methyl 2-(4-nitrophenyl)-2-(thiazol-2-yl)acetate** (**27**):

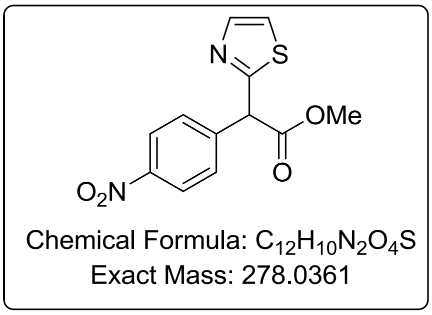


Isolated yield: 22 mg (36% 0.22 mmol scale), pale red oil; $R_f$: 0.35 (1/1, EtOAc/hexane); $^1$H NMR (600 MHz, $CDCl_3$) δ/ppm 8.21–8.25 (m, 2H), 7.78 (d, *J* = 3.3 Hz, 1H), 7.61–7.65 (m, 2H), 7.36 (d, *J* = 3.3 Hz, 1H), 5.54 (s, 1H), 3.68 (s, 3H); $^{13}$C NMR (151 MHz, $CDCl_3$) δ/ppm 169.9, 165.1, 147.9, 143.3, 142.9, 129.9, 124.2, 120.7, 54.8, 53.4; LC-MS (MeCN), Rt. 2.77 min, m/z = 279.1 $[M+H]^+$. HR-MS ($^+$ESI-TOF) calculated for $C_{12}H_{11}N_2O_4S$ 279.0440, found 279.0439 (Δ = 0.4 ppm).

**Ethyl 2-(4-nitrophenyl)-2-(thiazol-2-yl)acetate** (**29**):

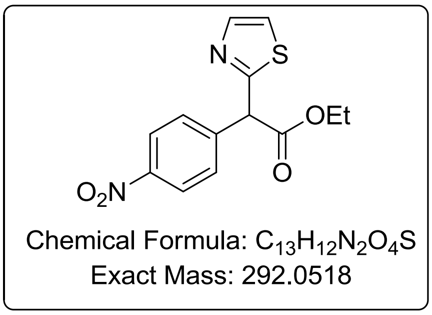


Isolated yield: 45 mg (35% 0.22 mmol scale), pale red oil; $R_f$: 0.85 (9/1, DCM/MeOH); $^1$H NMR (400 MHz, $CDCl_3$) δ/ppm 8.27–8.20 (m, 2H), 7.80 (d, *J* = 3.3 Hz, 1H), 7.69–7.62 (m, 2H), 7.38 (d, *J* = 3.3 Hz, 1H), 5.53 (s, 1H), 4.36–4.22 (m, 2H), 1.29 (t, *J* = 7.1 Hz, 3H); $^{13}$C NMR (101 MHz, $CDCl_3$) δ/ppm 169.4, 165.1, 147.7, 143.4, 142.8, 129.7, 124.1, 120.5, 62.5, 54.9, 14.0; IR (neat) ν = 3133 (w), 3079 (w), 2988 (w), 2944 (w), 2908

(w), 1733 (s), 1606 (m), 1597 (m), 1519 (s), 1493 (m), 1346 (s), 1322 (m), 1302 (m), 1234 (m), 1185 (s), 1156 (s), 1016 (m), 857 (m), 727 (m) 705 (m) $cm^{-1}$; LC-MS (MeCN), Rt. 2.77 min, m/z = 291.9 $[M-H]^-$. HR-MS ($^-$ESI-TOF) calculated for $C_{13}H_{11}N_2O_4S$ 291.0434, found 291.0440 (Δ = 2.1 ppm).

**2-Phenyl-2-(thiazol-2-yl)acetonitrile (31):**

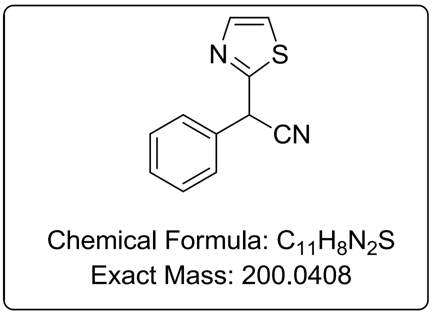


Isolated yield: 64 mg (73% 0.44 mmol scale), colourless oil; $R_f$: 0.20 (2/8, EtOAc/hexane); $^1$H NMR (600 MHz, $CDCl_3$) δ/ppm 7.79 (d, *J* = 3.3 Hz, 1H), 7.53–7.46 (m, 2H), 7.46–7.37 (m, 3H), 7.34 (d, *J* = 3.3 Hz, 1H), 5.56 (s, 1H); $^{13}$C NMR (151 MHz, $CDCl_3$) δ/ppm 164.3, 143.4, 133.5, 129.6, 129.3, 127.9, 121.2, 117.6, 41.2; IR (neat) ν = 3119 (w), 3088 (w), 3033 (w), 2982 (w), 2901 (w), 2367 (w), 2250 (w), 1494 (m), 14555 (m), 1129 (w), 1090 (w), 732 (s), 696 (s) $cm^{-1}$; LC-MS (MeOH), Rt. 2.65 min, m/z = 201.0 $[M+H]^+$. HR-MS ($^+$ESI-TOF) calculated for $C_{11}H_8N_2S$ 200.0486, found 200.0496 (Δ = 5.0 ppm).

**Methyl 3-phenyl-2-(thiazol-2-yl)propanoate (33):**

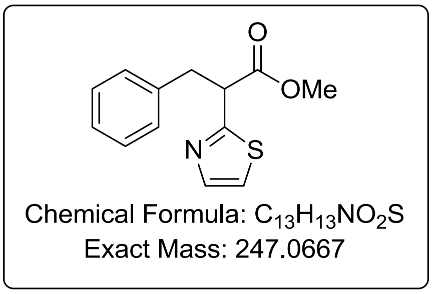


Isolated yield: 11 mg (16%, 0.22 mmol scale), pale yellow oil; Rf: 0.07 (1/9, EtOAc/hexane); $^1$H NMR (400 MHz, $CDCl_3$) δ/ppm 7.75 (d, *J* = 3.3 Hz, 1H), 7.29 –7.12 (m, 6H), 4.44 (dd, *J* = 8.5, 7.0 Hz, 1H), 3.67 (s, 3H), 3.47 (dd, *J* = 13.7, 8.5 Hz, 1H), 3.33 (dd, *J* = 13.7, 7.0 Hz, 1H); $^{13}$C NMR (101 MHz, $CDCl_3$) δ/ppm 171.6, 166.7, 142.4, 137.7, 129.0, 128.7, 127.0, 119.8, 52.7, 51.7, 39.7; IR (neat) ν = 3030.6 (w), 2147.6 (w), 1983.2 (w), 1747.7 (s), 1604.1 (w), 1496.4 (w), 1455.4 (w), 1436.2 (w), 1283.4 (m), 1162.6 (s), 1104.8 (m), 1081.8 (m), 1048.3 (m), 974.1 (m), 840.1 (w), 741.4 (m), 699.3 (s), 666.1 (m), 651.8 (m), 623.6 (m) $cm^{-1}$; LC-MS (MeCN), Rt. 0.28 min, m/z = 247.9 $[M+H]^+$, HR-MS ($^+$ESI-TOF) calculated for $C_{13}H_{14}NO_2S$ 248.0745, found 248.0749 (Δ = 1.6 ppm).

**Ethyl 3-phenyl-2-(thiazol-2-yl)propanoate (35):**

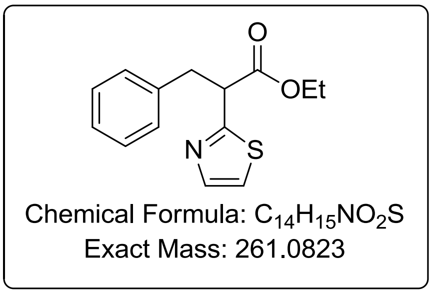


Isolated yield: 41 mg (36%, 0.44 mmol scale), colourless oil; $^1$H NMR (400 MHz, $CDCl_3$) δ/ppm 7.83 (d, *J* = 3.2 Hz, 1H), 7.34 (d, *J* = 3.3 Hz, 1H), 7.30–7.22 (m, 5H), 4.40 (s, 1H), 4.32–4.17 (m, 2H), 3.75 (d, *J* = 13.8 Hz, 1H), 3.42 (d, *J* = 13.8 Hz, 1H), 1.26 (t, *J* = 7.1 Hz, 3H); $^{13}$C NMR (101 MHz, $CDCl_3$) δ/ppm 171.9, 171.9, 143.1, 134.7, 130.5, 128.1, 127.2, 120.4, 79.2, 63.4, 45.0, 14.1; IR (neat) ν = 2978 (w), 2960 (w), 2181 (w), 2034 (w), 1686 (w), 1495 (w), 1453 (m), 1397 (w), 1261 (m), 1179 (m), 1152 (m), 1101 (m), 1030 (m), 860 (w), 732 (m), 698 (m) $cm^{-1}$; LC-MS (MeCN), Rt. 3.09 min, m/z = 262.1 $[M+H]^+$, HR-MS ($^+$ESI-TOF) calculated for $C_{14}H_{16}NO_2S$ 262.0913, found 262.0902 (Δ = 4.2 ppm).

**3-Phenyl-2-(thiazol-2-yl)propanamide (37):**

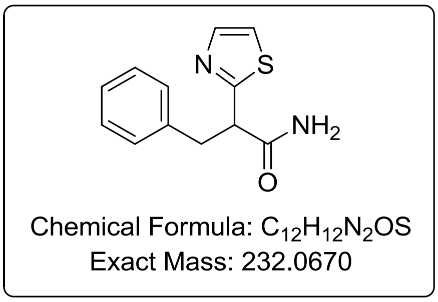


Isolated yield: 35 mg (34%, 0.44 mmol scale), amorphous white solid; $^{1}$H NMR (600 MHz, DMSO-$d_6$) δ/ppm 7.67 (d, *J* = 3.0 Hz, 1H), 7.65 (br, 1H) 7.59 (d, *J* = 3.3 Hz, 1H), 7.27–7.19 (m, 2H), 7.16 (m, 3H), 7.06 (br, 1H), 4.30 (dd, *J* = 8.7, 6.7 Hz, 1H), 3.26 (dd, *J* = 13.6, 8.7 Hz, 1H), 3.07 (dd, *J* = 13.6, 6.7 Hz, 1H); $^{13}$C NMR (101 MHz, $CDCl_3$) δ/ppm 172.3, 168.7, 142.0, 139.0, 129.3, 128.6, 126.7, 120.7, 51.9, 39.8; IR (neat) ν = 3291 (br), 3117 (br), 2918 (w), 1685 (s), 1680 (s), 1676 (s), 1497 (m), 1452 (w), 1428 (w), 1405 (m), 1315 (w), 1286 (w), 1251 (w), 1128 (s), 1061 (m), 835 (m), 745 (m), 728 (s), 695 (s); LC-MS (MeCN), Rt. 2.13 min, m/z = 233.4 $[M+H]^+$. HR-MS ($^+$ESI-TOF) calculated for $C_{12}H_{13}N_2OS$ 233.0749, found 233.0752 (Δ = 1.3 ppm); Melting point: 145 °C (decomposed, EtOH).

**3-(4-Bromophenyl)-2-(thiazol-2-yl)propanamide (39):**

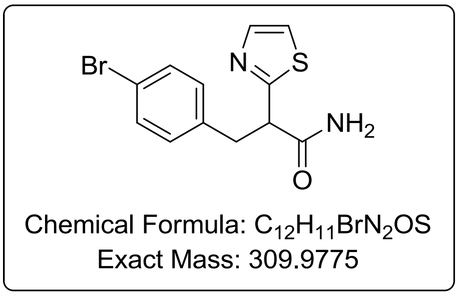


Isolated yield: 20 mg (37%, 0.176 mmol scale), White amorphous solid; $^{1}$H NMR (600 MHz, DMSO-$d_6$) δ/ppm 7.67 (d, *J* = 3.0 Hz, 1H), 7.65 (br, 1H), 7.59 (d, *J* = 3.3 Hz, 1H), 7.42 (d, *J* = 8.0 Hz, 2H), 7.12 (d, *J* = 8.0 Hz, 2H), 7.08 (s, 1H), 4.27 (dd, *J* = 8.9, 6.5 Hz, 1H), 3.22 (dd, *J* = 13.6, 8.7 Hz, 1H), 3.06 (dd, *J* = 13.6, 6.8 Hz, 1H); $^{13}$C NMR (151 MHz, DMSO-$d_6$) δ/ppm 172.1, 168.4, 142.1, 138.4, 131.6, 131.4, 120.8, 119.9, 51.7, 39.1; IR (neat) ν = 3303 (br), 3190 (br), 1681 (s), 1498 (m), 1486 (m), 1404 (m), 1284 (w), 1253 (w), 1131 (m), 1065 (m), 1011 (s), 847 (s), 797 (m), 736 (s), 606 (m), 537 (s) $cm^{-1}$; LC-MS (MeOH), Rt. 2.45 min, m/z = 310.9 $[M+H]^+$. HR-MS ($^+$ESI-TOF) calculated for $C_{12}H_{12}BrN_2OS$ 310.9854, found 310.9857 (Δ = 1.0 ppm); Melting point: 144 °C (decomposed, EtOH).

**Ethyl 2-(thiazol-2-yl)propanoate (41):**

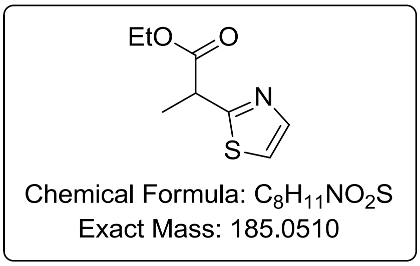


Isolated yield: 14 mg (35%, 0.22 mmol scale), pale yellow oil; Rf: 0.46 (1/1, EtOAc/hexane); $^{1}$H NMR (700 MHz, $CDCl_3$) δ/ppm 7.73 (d, *J* = 3.3 Hz, 1H), 7.29 (d, *J* = 3.3 Hz, 1H), 4.24–4.17 (m, 3H), 1.67 (d, *J* = 7.3 Hz, 3H), 1.26 (t, *J* = 7.1 Hz, 3H); $^{13}$C NMR (176 MHz, $CDCl_3$) δ/ppm 172.3, 168.8, 142.3, 119.4, 61.6, 44.4, 18.5, 14.2; IR (neat) ν = 3116.0 (w), 2983.1 (w), 2936.5 (w), 1731.6 (s), 1499.4 (w), 1446.4 (w), 1369.5 (w), 1254.2 (m), 1172.8 (m), 1137.6 (m), 1095.8 (m), 1046.7 (m), 1016.5 (m), 939.1 (w), 859 (w), 730.2 (m), 623.9 (w) $cm^{-1}$; LC-MS (MeCN), Rt. 2.35 min, m/z = 186.9 $[M+H]^+$. HR-MS ($^+$ESI-TOF) calculated for $C_8H_{12}NO_2S$ 186.0589, found 186.0588 (Δ = 0.5 ppm).

**Ethyl 2-(thiazol-2-yl)butanoate (43):**

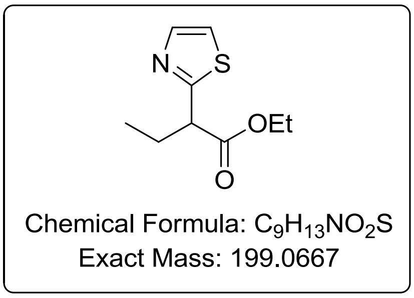


Isolated yield: 15 mg (33%, 0.66 mmol scale), colourless oil; $^{1}$H NMR (400 MHz, $CDCl_3$) δ/ppm: 7.73 (d, *J* = 3.3 Hz, 1H), 7.29 (dd, *J* = 3.3, 0.5 Hz, 1H), 4.27–4.14 (m, 2H), 4.04 (t, *J* = 7.5 Hz, 1H), 2.25–1.97 (m, 2H), 1.26 (t, *J* = 7.1 Hz, 3H), 0.97 (t, *J* = 7.4 Hz, 3H); $^{13}$C NMR (101 MHz, $CDCl_3$) δ/ppm: 171.9, 167.7, 142.3, 119.5, 61.5, 51.8, 27.5, 14.3, 11.9; IR (neat) ν = 3116 (w), 3085 (w), 2972 (w), 2937 (w), 2828 (w), 1733 (s), 1498 (w), 1461 (w), 1370 (w), 1253 (w), 1181 (s), 1138 (w), 1022 (m), 861 (w), 727 (w); LC-MS (MeCN), Rt. 2.48 min, m/z = 200.8 $[M+H]^+$; HR-MS ($^+$ESI-TOF) calculated for $C_9H_{14}NO_2S$ 200.0745, found 200.0751 (Δ = 3.0 ppm).

## Synthesis and spectroscopic data of starting materials.

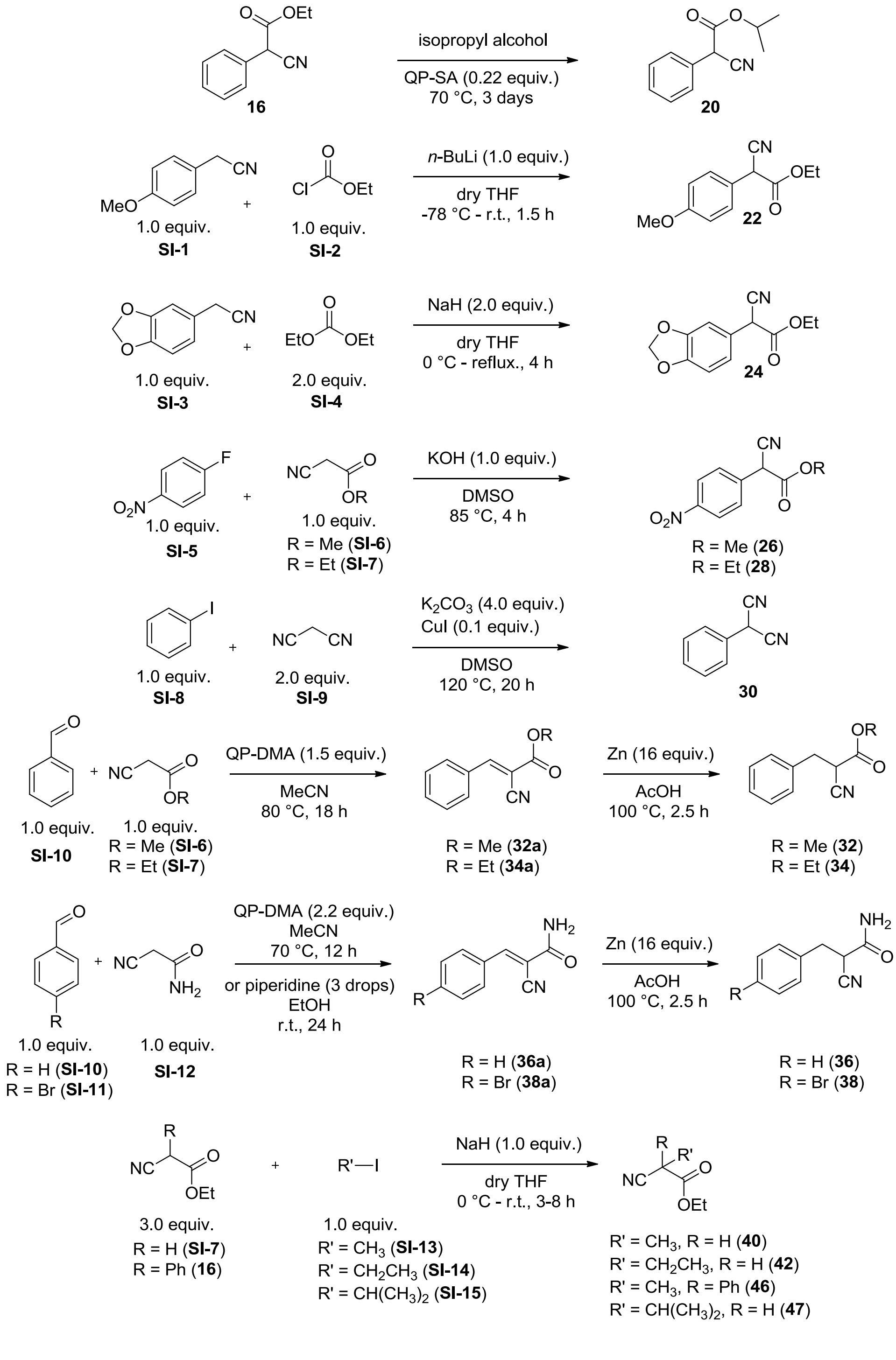


**Scheme S1**: Synthesis of starting materials.

**Isopropyl 2-cyano-2-phenylacetate (20):**

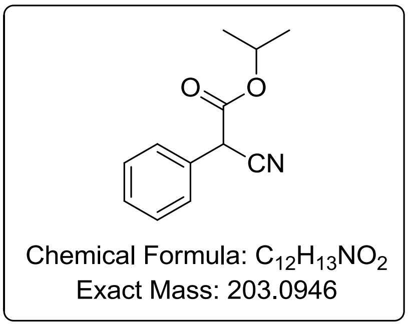


Ethyl phenylcyanoacetate (**16**, 0.473 g, 2.5 mmol) was dissolved in isopropyl alcohol (10 mL) and left stirring for 1 min. Polymer-bound sulfonic acid (QP-SA) (200 mg, 2.2 mmol/g loading) was then added and the mixture was stirred for 3 days at 70 °C. The QP-SA resin was filtered, and the solvent evaporated to give the product as a colourless oil which was used without any further purification. Isolated yield: 0.322 g (64% 2.5 mmol), colourless oil; $^{1}H$ NMR (400 MHz, $CDCl_3$) δ/ppm 7.51–7.40 (m, 5H), 5.07 (hept, *J* = 6.3 Hz, 1H), 4.70 (s, 1H), 1.28 (dd, *J* = 7.6, 6.3 Hz, 6H); $^{13}C$ NMR (101 MHz, $CDCl_3$) δ/ppm 164.6, 130.3, 129.4, 129.3, 128.0, 116.0, 71.6, 44.2, 21.6, 21.5; IR (neat) ν = 3069 (w), 3038 (w), 2986 (w), 2937 (w), 2253 (w), 1739 (s), 1601 (w), 1498 (w), 1467 (w), 1456 (m), 1377(w), 1353 (w), 1256 (m), 1235 (m), 1199 (m), 1101 (s), 1032 (w), 1016 (w), 1004 (w), 956 (w), 917 (w), 901 (w), 83 (w), 732 (m), 695 (s) $cm^{-1}$; LC-MS (MeCN), Rt. 2.93 min, m/z = 204.0 $[M+H]^{+}$. HR-MS ($^{+}$ESI-TOF) calculated for $C_{12}H_{14}NO$ 204.1025, found 204.1032 (Δ = 3.4 ppm).

**Ethyl 2-cyano-2-(4-methoxyphenyl)acetate (22):**

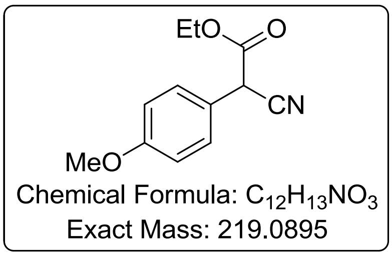


General procedure:[2]

4-Methoxyphenylacetonitrile (0.7 g, 4.76 mmol, 1.00 equiv) was dissolved in dry THF (4.7 mL) and cooled to −78 °C. At this temperature *n*-butyllithium (3.0 mL, 4.76 mmol, 1.00 equiv) was added dropwise and the colour of the solution changed immediately to yellow. After further stirring for 20 min at −78 °C, a solution of ethyl chloroformate (**SI-2**) (0.52 g, 4.76 mmol, 1.00 equiv) in THF (1 mL) was added slowly to the stirring solution. The reaction mixture stirred for 30 min at this temperature and was then allowed to warm to room temperature and stirred for another 1 h. The reaction was quenched with water and EtOAc was added. The layers were separated and the aqueous phase was extracted three times with EtOAc. The combined organic layer was washed twice with brine, dried over $Na_2SO_4$ and the solvent was removed under reduced pressure. The crude material was further purified on a silica column 1:9–4:6 EtOAc/hexane gradient to give the product. Isolated yield: 0.54 g (52%, 4.76 mmol scale), colourless oil; $R_f$: 0.32 (2/8, EtOAc/hexane). $^{1}H$ NMR (400 MHz, DMSO-$d_6$) δ/ppm 7.40–7.34 (m, 2H), 6.95–6.89 (m, 2H), 4.66 (s, 1H), 4.23 (qd, J = 7.1, 2.1 Hz, 2H), 3.81 (s, 3H), 1.27 (t, J = 7.1 Hz, 3H); $^{13}C$ NMR (101 MHz, DMSO-$d_6$) δ/ppm 165.4, 160.3, 129.2, 122.0, 116.0, 114.8, 63.3, 55.5, 43.1, 14.0; IR (neat) ν = 3743.6 (w), 2984.0 (w), 2940.0 (w), 2910.0 (w), 2839.3 (w), 2248.0 (w), 2000.6 (w), 1741.4 (m), 1610.8 (m), 1586.2 (w), 1509.5 (s), 1463.6 (w), 1443.4 (w), 1422.9 (w), 1368.5 (w), 1303.8 (m), 1247.1 (s), 1198.8 (m), 1178.8 (m), 1111.8 (w), 1095.3 (w), 1027.1 (m), 942. (w), 833.5 (m), 811.2 (m), 793.1 (w), 752.7 (w), 633.8 (w) $cm^{-1}$; LC-MS (MeCN), Rt. 2.69 min, m/z = 218.2 $[M-H]^{-}$. HR-MS calculated for $C_{12}H_{14}NO_3$ 220.0974, found 220.0970 (Δ = 1.8 ppm).

[2] Doraswamy, K.; Venkata Ramana, P. *Chem. Sci. Trans.*, **2013**, *2*, 649-655.

**Ethyl 2-(benzo[*d*][1,3]dioxol-5-yl)-2-cyanoacetate (24):**

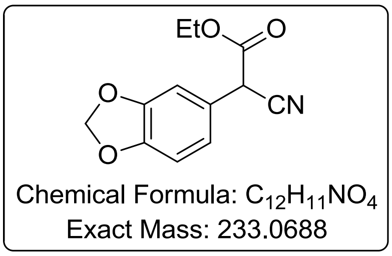


General procedure:[3]

Sodium hydride (0.799 g, 20.0 mmol) was slowly added to solution of 3,4-(methylenedioxy)phenylacetonitrile (1.61 g, 10.0 mmol) in THF (20 mL) at 0 °C. Diethyl carbonate (2.36 g, 20.0 mmol) was then added dropwise at room temperature and the mixture was refluxed for 4 h. The reaction was then quenched with 2 M HCl (20 mL) and most of the THF removed using a rotatory evaporator. The residue was then extracted with EtOAc (3 × 20 mL), the organic layer was dried over $Na_2SO_4$, filtered and solvent evaporated under reduced pressure to obtain an orange oil which was purified using column chromatography (80:20, hexanes/EtOAc). Isolated yield: 1.74 g (75%, 10.0 mmol scale), colourless oil; $^1$H NMR (400 MHz, $CDCl_3$) δ/ppm 6.94–6.89 (m, 2H), 6.82 (dd, *J* = 7.7, 0.7 4Hz, 1H), 6.01 (s, 2H), 4.61 (s, 1H), 4.25 (qd, *J* = 7.1, 2.2 Hz, 2H), 1.29 (t, *J* = 7.1 Hz, 3H); $^{13}$C NMR (101 MHz, $CDCl_3$) δ/ppm 165.2, 148.6, 148.6, 123.5, 121.9, 115.8, 108.9, 108.4, 101.8, 63.5, 43.5, 14.1; IR (neat) ν = 2986 (w), 2941 (w), 2906 (w), 1744 (s), 1504 (s), 1491 (s), 1447 (m), 1369 (w), 1251 (s), 1105 (w), 1038 (s), 934 (w) $cm^{-1}$; LC-MS (MeCN), Rt. 2.67 min, m/z = 233.9 $[M+H]^+$. HR-MS ($^+$ESI-TOF) calculated for $C_{12}H_{12}NO_4$ 234.0758, found 234.0766 (Δ = 3.4 ppm).

**Methyl 2-cyano-2-(4-nitrophenyl)acetate (26):**

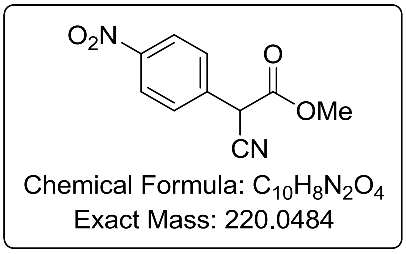


Potassium hydroxide (0.40 g, 7.09 mmol, 1.00 equiv) was stirred in DMSO (5 mL) to form a homogeneous solution in a sealed microwave vial. After 10 min of stirring in a water bath at 85 °C methyl 2-cyanoacetate **SI-6** (1.51 mL, 7.09 mmol, 1.00 equiv) was added by means of a syringe and the solution was stirred for a further 30 min. Then a solution of 1-fluoro-4-nitrobenzene **SI-5** (2.00 g, 7.09 mmol, 1.00 equiv) in DMSO (2 mL) was added dropwise at 85 °C. After 4 h the reaction mixture was poured in a beaker filled with ice and 1 M HCl (5 mL). The vial was washed with EtOAc (20 mL) and the resulting solution was allowed to stir for 2 h. The two layers were separated and the aqueous layer was extracted with EtOAc (3 × 25 mL). The combined organic layers were washed twice with brine, dried over $Na_2SO_4$ and the solvent was removed under reduced pressure. The crude material was further purified on a silica column with pure hexanes as eluent to remove **SI-5**. Methyl 2-cyanoacetate was removed on high vacuum. Isolated yield: 0.834 g (53%, 7.09 mmol scale), red solid; $R_f$: 0.05 (2/8, EtOAc/hexane); $^1$H NMR (700 MHz, $CDCl_3$) δ/ppm 8.28 (d, *J* = 8.8 Hz, 2H), 7.67 (d, *J* = 8.7 Hz, 2H), 4.87 (s, 1H), 3.83 (s, 3H); $^{13}$C NMR (176 MHz, $CDCl_3$) δ/ppm 164.2, 148.5, 136.3 129.2, 124.5, 114.4, 54.4, 43.1; IR (neat) ν = 3133 (w), 3086 (w), 2973 (w), 2937 (w), 1740 (s), 1735 (s), 1610 (w), 1597 (w), 1523 (s), 1437 (m), 1348 (s), 1313 (m), 1289 (s), 1234 (s), 1165 (m), 1107 (m), 857 (m), 779 (m), 736 (s) $cm^{-1}$; LC-MS (MeCN), Rt. 2.44 min, m/z = 218.92 $[M-H]^-$. HR-MS calculated for $C_{10}H_7N_2O_4$ 219.0406, found 219.0409 (Δ = 1.4 ppm); Melting point: 93–95 °C.

[3] Nappi, M.; Bergonzini, G.; Melchiorre, P. *Angew. Chem. Int. Ed.* **2014**, *53*, 4921-4925.

**Ethyl 2-cyano-2-(4-nitrophenyl)acetate (28)**

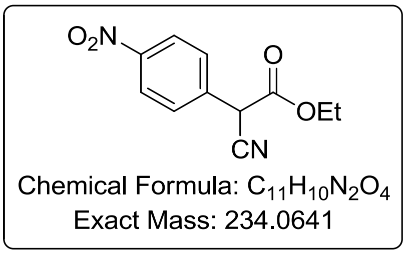


Potassium hydroxide (0.80 g, 14.18 mmol, 1.00 equiv) was stirred in DMSO (10 mL) to a homogeneous solution in a sealed microwave vial. After 10 min of stirring in a water bath at 85 °C ethyl 2-cyanoacetate **SI-7** (1.51 mL, 14.18 mmol, 1.00 equiv) was added by means of a syringe and the solution was stirred for further 30 min. Then a solution of 1-fluoro-4-nitrobenzene **SI-5** (2.00 g, 14.18 mmol, 1.00 equiv) in DMSO (4 mL) was added dropwise at 85 °C. After 4 h a TLC showed complete conversion and the mixture was transferred in a beaker prepared with ice and 1 M HCl (10 mL). The vial was washed with EtOAc (40 mL) and the resulting solution was allowed to stir for 2 h. The two layers were separated and the aqueous layer was extracted three times with EtOAc. The combined organic layers were washed twice with brine, dried over $Na_2SO_4$ and the solvent was removed under reduced pressure. For the final purification a silica column with pure hexane as eluent was used to remove **SI-5**. Ethyl 2-cyanoacetate was removed on high vacuum. Isolated yield: 1.89 g (57% 14.18 mmol scale), red oil; $R_f$: 0.17 (1/1, EtOAc/hexane); $^1$H NMR (700 MHz, $CDCl_3$) δ/ppm 8.28–8.32 (m, 2H), 7.66–7.70 (m, 2H), 4.84 (s, 1H), 4.29 (qd, *J* = 7.1, 1.4 Hz, 2H), 1.31 (t, *J* = 7.1 Hz, 3H); $^{13}$C NMR (176 MHz, $CDCl_3$) δ/ppm 163.8, 148.6, 136.6, 129.3, 124.6, 114.6, 64.2, 43.5, 14.0; IR (neat) ν = 3733 (w), 3588 (w), 2988 (w), 2205 (w), 2059 (w), 2032 (w), 2016 (w), 1999 (w), 1967 (w), 1742 (s), 1608 (w), 1522 (s), 1494 (w), 1468 (w), 1446 (w), 1346 (s), 1318 (m), 1297 (m), 1241 (m), 1199 (m), 1157 (m), 1109 (m), 1016 (m), 855 (m), 828 (m), 770 (w), 734 (m), 692 (m) $cm^{-1}$; LC-MS (MeCN), Rt. 2.69 min, m/z = 233.2 $[M-H]^-$. HR-MS calculated for $C_{11}H_{11}N_2O_4$ 235.0719, found 235.0725 (Δ = 2.6 ppm).

**Ethyl 2-cyano-2-(4-nitrophenyl)acetate (30)**

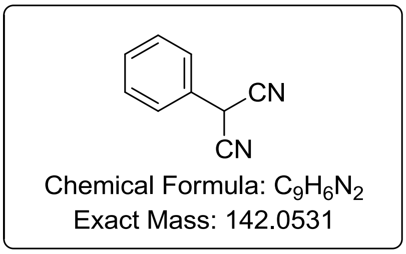


General procedure:[4]

Malonitrile **SI-9** (1.06 g, 16.00 mmol, 2.00 equiv) and iodobenzene **SI-8** (1.63 g, 8.00 mmol, 1.00 equiv) were stirred in DMSO (20 mL) with copper iodide (0.152 g, 0.8 mmol, 0.1 equiv) and potassium carbonate (4.40 g, 32.00 mmol, 4 equiv). The reaction mixture was heated to 120 °C. After 20 h the reaction mixture was poured in a beaker with 1 M HCl (15 mL). The resulting mixture was filtered through silica and extracted with EtOAc (50 mL). The two layers were separated and the organic layer was washed five times with brine (5 × 250 mL). The combined organic layers were dried over $Na_2SO_4$ and the solvent was removed under reduced pressure. The crude product was then purified using a silica column with EtOAc/hexane as eluent to give the product as a white solid. Isolated yield: 0.455 g (40% 8.00 mmol scale), white solid; $R_f$: 0.26 (2/8, EtOAc/hexane); $^1$H NMR (600 MHz, $CDCl_3$) δ/ppm 7.54–7.47 (m, 5H), 5.08 (s, 1H); $^{13}$C NMR (151 MHz, $CDCl_3$) δ/ppm 130.4, 130.0, 127.2, 126.2, 111.7, 28.1; IR (neat) ν = 3656 (w), 2982 (br), 2889 (br), 2257 (w), 1495 (s), 1455 (m), 1134 (br), 1014 (w), 1004 (w), 752 (m), 734 (s), 694 (s) $cm^{-1}$; LC-MS (MeOH), Rt. 2.57 min, m/z = 140.1 $[M-H]^-$. HR-MS calculated for $C_9H_5N_2$ 141.0453, found 141.0458 (Δ = 3.5 ppm); Melting point: 67-68 °C (EtOH).

[4] Okuro, K.; Furuune, M.; Miura, M.; Nomura, M. *J. Org, Chem.* **1993**, *58*, 7606-7607.

**(*E*)-Methyl-2-cyano-3-phenylacrylate (32a):**

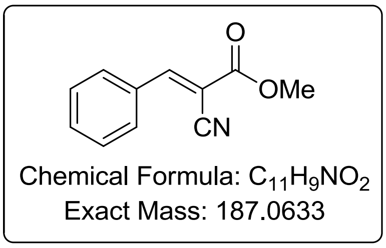


Benzaldehyde **SI-10** (1.59 g, 15 mmol, 1.0 equiv) and methyl 2-cyanoacetate **SI-6** (1.59 g, 16 mmol 1.1 equiv) were dissolved in 50 mL acetonitrile and polymer bound dimethyl benzylamine (QP-DMA, 10 g) were added. The whole mixture was stirred under $N_2$ at 80 °C for 18 h. The solvent was then removed under reduced pressure and the crude solid was recrystallised from ethanol to give 1.64 g of white crystals. Isolated yield: 1.64 g (58%, 15 mmol), white crystals; Rf: 0.33 (2/8, EtOAc/hexane); $^1$H NMR (700 MHz, $CDCl_3$) δ/ppm 8.27 (s, 1H), 8.01–7.98 (m, 2H), 7.59–7.55 (m, 1H), 7.53–7.49 (m, 2H), 3.94 (s, 3H); $^{13}$C NMR (176 MHz, $CDCl_3$) δ/ppm 163.3, 155.6, 133.7, 131.7, 131.4, 129.5, 115.7, 102.8, 53.7; IR (neat) ν = 2358 (s), 2341 (s), 1654 (w), 668 (s) $cm^{-1}$; LC-MS (MeCN), Rt. 2.84 min, m/z = 188.1 $[M+H]^+$. HR-MS ($^+$ESI-TOF) calculated for $C_{11}H_{10}NO_2$ 188.0712, found 188.0716 (Δ = 2.1 ppm); Melting point: 89-90 °C (EtOH). Lit.: 89 °C (EtOH).[5]

**Methyl-2-cyano-3-phenylpropanoate (32):**

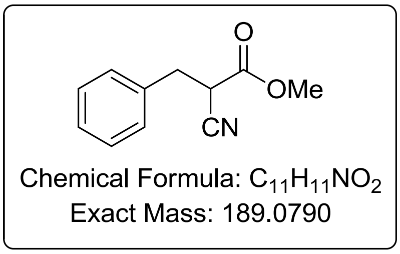


(*E*)-methyl-2-cyano-3-phenylacrylate **30a** (1.40 g, 7.48 mmol, 1.0 equiv) and zinc powder (7.83 g, 119.7 mmol, 16.0 equiv) were dissolved in 50 mL glacial acetic acid and the solution was stirred for 2.5 h at 100 °C. The reaction mixture was filtered through a celite pad and washed with 100 mL EtOAc. The filtrate was neutralised with aq. $NaHCO_3$ solution and the organic layer was separated, washed with brine (2 × 25 mL), dried over $Na_2SO_4$ and the solvent removed under reduced pressure. The crude product was further purified on a silica column with a solvent mixture of 1:9 (EtOAc/hexane) with the desired nitrile isolated as a colourless oil.[6] Isolated yield: 0.583 g (41%, 7.48 mmol), colourless oil; Rf: 0.44 (1/9, EtOAc/hexane); $^1$H NMR (700 MHz, $CDCl_3$) δ/ppm 7.36–7.32 (m, 2H), 7.31–7.28 (m, 1H), 7.26 (dd, $J$ = 7.0, 1.6 Hz, 2H), 3.78 (s, 3H), 3.73 (ddd, $J$ = 8.5, 5.7, 0.9 Hz, 1H), 3.27 (dd, $J$ = 13.9, 5.7 Hz, 1H), 3.19 (dd, $J$ = 13.9, 8.5 Hz, 1H); $^{13}$C NMR (176 MHz, $CDCl_3$) δ/ppm 166.1, 135.3, 129.1, 128.9, 127.9, 116.1, 53.6, 39.6, 35.8; IR (neat) ν = 3032 (w), 2956 (w), 2360 (w), 2250 (w), 1743 (s), 1604 (w), 1585 (w), 1497 (m), 1455 (s), 1435 (s), 1340 (m), 1310 (m), 1263 (s), 1210 (s), 1190 (s), 1166 (s), 1081 (m), 1028 (s), 964 (w), 910 (w), 860 (w), 819 (w), 799 (w), 747 (s), 699 (s), 641 (m) $cm^{-1}$; LC-MS (MeCN), Rt. 2.60 min, m/z = 189.9 $[M+H]^+$. HR-MS ($^+$ESI-TOF) calculated for $C_{11}H_{12}NO_2$ 190.0868, found 190.0877 (Δ = 4.7 ppm).

**(*E*)-Ethyl-2-cyano-3-phenylacrylate (34a):**

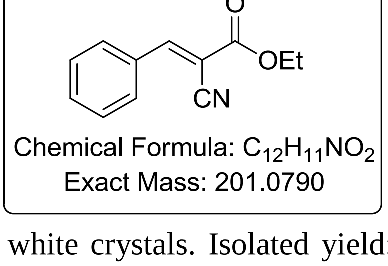


Freshly distilled benzaldehyde **SI-10** (1.59 g, 15 mmol, 1.0 equiv) and ethyl 2-cyanoacetate **SI-7** (1.81 g, 16 mmol 1.1 equiv) were dissolved in acetonitrile (50 mL) and QP-DMA (10 g) was added. The mixture was stirred under $N_2$ at 80 °C for 18 h; the QP-DMA was filtered off and the solvent removed under reduced pressure to give the crude solid which was recrystallised from ethanol to give the desired nitrile as white crystals. Isolated yield: 1.166 g (39%, 15 mmol scale), white crystals; $R_f$: 0.35 (1/9,

[5] Saeed, B.; Morteza, B.; Shohreh, H.; Peyman, S. *Syn. Commun.* **2006**, *36*, 2549-2557.
[6] Nath, D.; Skilbeck, M. C.; Coldham, I.; Fleming, F. F. *Org. Lett.* **2014**, *16*, 62-65.

EtOAc/hexane); $^1$H NMR (700 MHz, $CDCl_3$) δ/ppm 8.25 (s, 1H), 7.99 (dd, *J* = 7.4, 1.7 Hz, 2H), 7.54–7.58 (m, 1H), 7.48–7.53 (m, 2H), 4.39 (q, *J* = 7.2 Hz, 2H), 1.40 (t, *J* = 7.2 Hz, 3H); $^{13}$C NMR (176 MHz, $CDCl_3$) δ/ppm 162.6, 155.2, 133.4, 131.6, 131.2, 129.4, 115.6, 103.2, 62.9, 14.3; IR (neat) ν = 3924 (w), 3869 (w), 3734 (w), 2568 (w), 2366 (s), 2203 (w), 2165 (w), 2033 (w), 1993 (w), 1963 (w) $cm^{-1}$; LC-MS (MeCN), Rt. 2.88 min, m/z = 202.1 $[M+H]^+$. HR-MS ($^+$ESI-TOF) calculated for $C_{12}H_{12}NO_2$ 202.0868, found 202.0865 (Δ = 1.5 ppm); Melting point: 49–50 °C. (Literature: 49-50 °C).

**Ethyl 2-cyano-3-phenylpropanoate (34):**

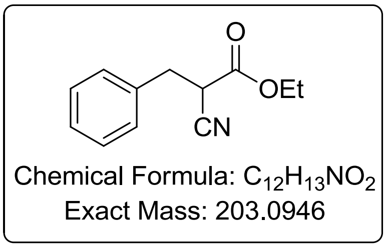


(*E*)-Ethyl-2-cyano-3-phenylacrylate **34a** (0.80 g, 3.98 mmol) and zinc powder (3.98 g, 60.8 mmol, 16.0 equiv) were dissolved in 25 mL glacial acetic acid and the solution was stirred for 2.5 h at 100 °C. The reaction mixture was filtered through a celite pad and washed with EtOAc (50 mL). The filtrate was neutralised with aq. $NaHCO_3$ solution and the organic layer was separated, washed with brine (2 × 25 mL), dried over $Na_2SO_4$ and the solvent removed under reduced pressure to obtain the an oil which was further purified on a short silica column with a solvent mixture of 1:9 (EtOAc/hexane). Isolated yield: 0.53 g (56%, 3.98 mmol scale), pale yellow oil; $^1$H NMR (700 MHz, $CDCl_3$) δ/ppm 7.32–7.37 (m, 2H), 7.30–7.32 (m, 1H), 7.26–7.29 (m, 2H), 4.24 (qd, *J* = 7.1, 1.8 Hz, 2H), 3.72 (ddd, *J* = 8.4, 5.8, 1.1 Hz, 1H), 3.28 (dd, *J* = 13.9, 5.8 Hz, 1H), 3.20 (dd, *J* = 13.9, 8.5 Hz, 1H), 1.27 (t, *J* = 7.2 Hz, 3H); $^{13}$C NMR (176 MHz, $CDCl_3$) δ/ppm 165.6, 135.4, 129.1, 128.9, 127.9, 116.3, 63.1, 39.8, 35.9, 14.1; IR (neat) ν = 3837 (w), 3815 (w), 3768 (w), 2746 (w), 3677 (w), 3648 (w), 2982 (w), 2936 (w), 2248 (w), 1995 (w), 1974 (w), 1739 (s), 1604 (w), 1496 (w), 1454 (w), 1392 (w), 1369 (w), 1256 (m), 1196 (m), 1163 (m), 1095 (w), 1080 (w), 1028 (m), 856 (w), 746 (m), 698 (s) $cm^{-1}$; LC-MS (MeCN), Rt. 2.78 min, m/z = 204.1 $[M+H]^+$. HR-MS ($^+$ESI-TOF) calculated for $C_{12}H_{14}NO_2$ 204.1025, found 204.1019 (Δ = 2.9 ppm).

**(*E*)-2-Cyano-3-phenylacrylamide (36a):**

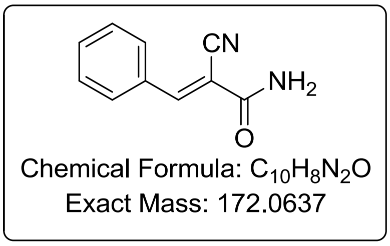


Benzaldehyde **SI-10** (1.06 g, 10.0 mmol) and cyanoacetamide (0.84 g, 10.0 mmol) were dissolved in ethanol (28 mL) to which 3 drops of piperidine were added and the solution was stirred for 24 h at rt. The solvent was then evaporated under reduced pressure to give a yellow solid which was recrystallised from ethanol. Isolated yield: 1.54 g (89%, 10.0 mmol scale), white amorphous solid; $^1$H NMR (400 MHz, DMSO-$d_6$) δ/ppm 8.19 (s, 1H), 8.02–7.86 (m, 3H), 7.79 (s, 1H), 7.62–7.52 (m, 3H); $^{13}$C NMR (101 MHz, $CDCl_3$) δ/ppm 163.1, 151.0, 132.8, 132.4, 130.5, 129.7, 116.9, 107.2; IR (neat) ν = 3395 (br), 3315 (w), 3151 (br), 2218 (w), 1685 (s), 1595 (m), 1573 (m), 1496 (w), 1494 (w), 1369 (m), 1355 (m), 1314 (w), 1290 (w), 1184 (m), 1104 (w), 765 (m), 741 (m), 683 (s), 677 (s), 595 (s), 582 (s) $cm^{-1}$; LC-MS (MeOH), Rt. 2.15 min, m/z = 173.0 $[M+H]^+$. HR-MS ($^+$ESI-TOF) calculated for $C_{10}H_9N_2O$ 173.0715, found 173.0717 (Δ = 1.2 ppm); Melting point: 120-121 °C (EtOH).

**2-Cyano-3-phenylpropanamide (36):**

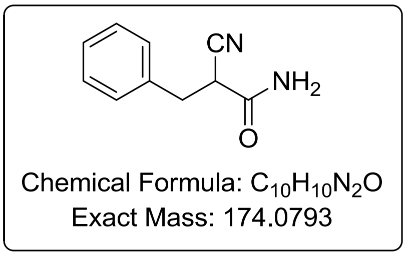


(*E*)-2-Cyano-3-phenylacrylamide **36a** (0.50 g, 2.9 mmol) and zinc powder (3.03 g, 46.4 mmol, 16.0 equiv) were dissolved in glacial acetic acid (12.5 mL) and the solution was stirred for 2.5 h at 100 °C. The reaction mixture was filtered through a celite pad and washed with 50 mL EtOAc. The filtrate was neutralised with aq. $NaHCO_3$ solution and the organic layer was separated, washed with brine (2 × 25 mL), dried over $Na_2SO_4$ and the solvent removed under reduced pressure to obtain a white powder which was recrystallised from ethanol to give the product as a white solid. Isolated yield: 0.432 g (86%, 2.9 mmol scale), white amorphous solid; $^1$H NMR (400 MHz, DMSO-$d_6$) δ/ppm 7.79 (s, 1H), 7.50 (s, 1H), 7.40–7.20 (m, 5H), 3.95 (dd, $J$ = 8.9, 6.5 Hz, 1H), 3.16 (dd, $J$ = 13.6, 6.5 Hz, 1H), 3.04 (dd, $J$ = 13.7, 8.9 Hz, 1H); $^{13}$C NMR (101 MHz, DMSO-$d_6$) δ/ppm 166.7, 137.3, 129.4, 128.9, 127.5, 118.9, 40.0, 35.7; IR (neat) ν = 3379 (br), 3314 (w), 3239 (w), 3196 (br), 3055 (w), 3032 (w), 2946 (w), 2920 (w), 2257 (w), 1663 (s), 1653 (s), 1622 (m), 1497 (m), 1457 (m), 1415 (m), 1349 (w), 1332 (w), 1283 (m), 1179 (m), 1133 (m), 1081 (w), 775 (m), 711 (s), 630 (s), 617 (s), 593 (s) $cm^{-1}$; LC-MS (MeCN), Rt. 2.10 min, m/z = 175.2 $[M+H]^+$. HR-MS ($^+$ESI-TOF) calculated for $C_{10}H_{11}N_2O$ 175.0871, found 175.0874 (Δ = 1.7 ppm); Melting point: 129-130 °C (EtOH).

**(*E*)-3-(4-Bromophenyl)-2-cyanoacrylamide (38a):**

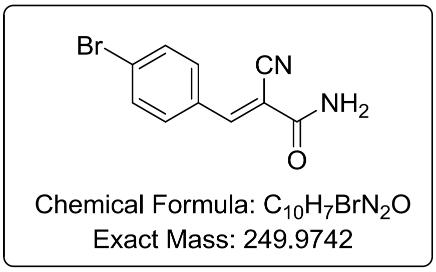


4-Bromobenzaldehyde **SI-11** (1.85 g, 10 mmol,) and cyanoacetamide **SI-12** (0.81 g, 10 mmol) were dissolved in acetonitrile (100 mL) and QP-DMA (10 g) was added. The mixture was stirred under $N_2$ at 70 °C for 12 h. The QP-DMA was filtered off and the solvent was removed under reduced pressure to give a yellow solid which was recrystallised from isopropyl alcohol. Isolated yield: 3.75 g, (60%, 10.0 mmol scale), yellow amorphous solid; $^1$H NMR (400 MHz, $CDCl_3$) δ/ppm 8.28 (s, 1H), 7.85–7.77 (m, 2H), 7.68–7.61 (m, 2H), 6.33 (s, 1H), 5.78 (s, 1H); $^{13}$C NMR (101 MHz, $CDCl_3$) δ/ppm 161.5, 152.8, 132.9, 132.1, 130.5, 128.2, 116.9, 103.7; IR (neat) ν = 3437 (m), 3302 (w), 3142 (m), 3056 (w), 2216 (w), 1696 (s), 1580 (s), 1601 (s), 1559 (m), 1489 (s), 1374 (s), 1309 (s), 1281 (m), 1206 (m), 1186 (m), 1125 (w), 1115 (m), 1006 (m), 828 (s), 809 (s), 777 (m), 697 (m), 577 (s) $cm^{-1}$; LC-MS (MeCN), Rt. 2.58 min, m/z = 251.0$[M+H]^+$. HR-MS ($^+$ESI-TOF) calculated for $C_{10}H_8BrN_2O$ 250.9820, found 250.9826 (Δ = 2.4 ppm); Melting point: 212-217 °C (decomposed, *i*PrOH).

**3-(4-Bromophenyl)-2-cyanopropanamide (38):**

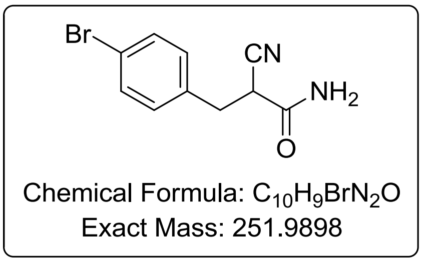


(*E*)-3-(4-Bromophenyl)-2-cyanoacrylamide **38a** (0.377 g, 1.50 mmol) and zinc powder (1.56 g, 24.0 mmol, 16.0 equiv) were dissolved in glacial acetic acid (8 mL) and the solution was stirred for 2.5 h at 100 °C. The reaction mixture was filtered through a celite pad and washed with EtOAc (30 mL). The filtrate was neutralised with sat. aq. $NaHCO_3$ solution and the organic layer was separated, washed with brine (2 × 15 mL), dried over $Na_2SO_4$ and the solvent removed under reduced pressure to obtain the crude product as an off white solid which was recrystallised from isopropyl alcohol. Isolated yield: 0.314 g (83%, 1.5 mmol scale), white amorphous solid; $^1$H NMR (400 MHz, $CDCl_3$) δ/ppm 7.79 (s, 1H), 7.60–7.47

(m, 3H), 7.30–7.23 (m, 2H), 3.95 (dd, $J$ = 8.7, 6.7 Hz, 1H), 3.13 (dd, $J$ = 13.7, 6.7 Hz, 1H), 3.05 (dd, $J$ = 13.7, 8.7 Hz, 1H); $^{13}$C NMR (101 MHz, $CDCl_3$) δ/ppm 166.5, 136.8, 131.8, 131.7, 120.8, 118.7, 39.7, 34.9; IR (neat) ν = 3391 (w), 3307 (w), 3192 (w), 2257 (w), 1685 (s), 1619 (w), 1493 (m), 1442 (w), 1401 (m), 1242 (w), 1197 (w), 1179 (w), 1074 (m), 1013 (m), 946 (w), 917 (w), 785 (s), 717 (w), 616 (s), 604 (s) $cm^{-1}$; LC-MS (MeCN), Rt. 2.72 min, m/z = 252.9 $[M+H]^+$. HR-MS ($^+$ESI-TOF) calculated for $C_{10}H_{10}BrN_2O$ 252.9976, found 252.9987 (Δ = 4.4 ppm); Melting point: 176 °C (decomposed, *i*PrOH).

**Ethyl 2-cyanopropanoate (40):**

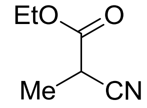


Chemical Formula: $C_6H_9NO_2$
Exact Mass: 127.0633

General procedure:[7]
Sodium hydride (58 mg, 1.46 mmol, 0.33 equiv, 60% in paraffin) was suspended in dry THF (1.46 mL) and stirred under $N_2$ at 0 °C. A solution of ethyl-2-cyano-acetate (500 mg, 4.42 mmol, 1.0 equiv) in THF (0.737 mL) was added using a syringe. After 15 min stirring at 0 °C, iodomethane (90.8 μL, 207 mg, 1.46 mmol, 0.33 equiv) was added and reaction left stirring for 3 h. The reaction mixture was then quenched with water (10 mL), diethyl ether (10 mL) added and the separated organic layer was washed with brine (3 × 25 mL). The organic layer was dried over $Na_2SO_4$ und the solvent was removed under reduced pressure. The product was then purified using a silica column with a solvent mixture of 5% (EtOAc/hexane) and the desired nitrile was recovered as a colourless liquid (51 mg, 83% yield). Isolated yield: 51 mg (83%, 1.46 mmol), colorless oil; Rf: 0.55 (1/1, EtOAc/hexane). $^1$H NMR (700 MHz, $CDCl_3$) δ/ppm 4.25 (q, $J$ = 7.2 Hz, 2H), 3.52 (q, $J$ = 7.4 Hz, 1H), 1.60–1.59 (d, $J$ = 7.4 Hz, 3H), 1.31 (t, $J$ = 7.2 Hz, 3H); $^{13}$C NMR (176 MHz, $CDCl_3$) δ/ppm 166.6, 117.5, 62.9, 31.6, 15.4, 14.1; IR (neat) ν = 3116.0 (w), 2983.1 (w), 2936.5 (w), 1731.6 (s), 1499.4 (w), 1446.4 (w), 1369.5 (w), 1254.2 (m), 1172.8 (m), 1095.8 (m), 1046.7 (m), 1016.5 (m), 939.1 (w), 859.0 (w), 730.2 (m), 623.9 (w) $cm^{-1}$; LC-MS (MeCN), Rt. 1.99 min, m/z = 227.8 $[M+H]^+$. HR-MS ($^+$ESI-TOF) calculated for $C_6H_{10}NO_2$ 128.0713, found 128.0713 (Δ = 0.8 ppm).

**Ethyl 2-cyanobutanoate (42):**

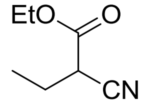


Chemical Formula: $C_7H_{11}NO_2$
Exact Mass: 141.0790

A solution of cyanoethyl acetate (2.172 g, 19.2 mmol) in 3 mL dry THF was slowly added to a suspension of sodium hydride (0.256 g, 6.4 mmol, 60% in paraffin) and THF (5 mL) under nitrogen atmosphere at 0 °C. After 15 min, a solution of iodoethane (0.998 g, 6.4 mmol) in THF (3 mL) was added dropwise and after stirring the suspension for 15 min at 0 °C, the reaction mixture was left to warm to room temperature and stirred for 8 h. The reaction was then quenched with 1 M HCl solution (10 mL) and extracted using EtOAc (2 × 25 mL). The organic layer was dried over sodium sulfate and solvent evaporated under reduced pressure to give the crude as an orange oil. The crude was purified on a silica column (1:4, EtOAc/hexanes) to give the desired. Isolated yield: 0.740 g (82%, 6.4 mmol), colourless oil; $^1$H NMR (400 MHz, $CDCl_3$) δ/ppm 4.27 (q, $J$ = 7.1 Hz, 2H), 3.46 (dd, $J$ = 7.4, 6.1 Hz, 1H), 2.07–1.92 (m, 2H), 1.33 (t, $J$ = 7.1 Hz, 3H), 1.13 (t, $J$ = 7.4 Hz, 3H); $^{13}$C NMR (101 MHz, $CDCl_3$) δ/ppm 166.2, 116.6, 62.9, 39.2, 23.8, 14.2, 11.4; IR (neat) ν = 2981 (w), 2941 (w), 2884 (w), 2249 (w), 1741 (s), 1462 (w), 1389 (w), 1370 (w), 1247 (m), 1189 (m), 1110 (w), 1021 (m), 955 (w), 854 (w), 811 (w) $cm^{-1}$;

[7] Yin, L.; Kanai, M.; Shibasaki, M. *Tetrahedron* **2012**, *68*, 3497-3506.

ASAP-MS (MeCN), Rt. 0.41 min, m/z = 142.1 $[M+H]^+$. HR-MS ($^+$ASAP) calculated for $C_7H_{12}NO_2$ 141.0862, found 141.0868 (Δ = 4.2 ppm).

**Ethyl 2-cyano-2-phenylpropanoate (46):**

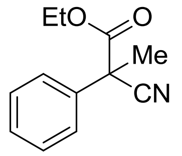


Chemical Formula: $C_{12}H_{13}NO_2$
Exact Mass: 203.0946

Ethyl-2-cyano-2-phenylacetate **16** (300 mg, 1.60 mmol, 1.0 equiv) was dissolved in 1.55 mL dry DMF and cooled to 0 °C under a $N_2$-atmosphere. Sodium hydride (70 mg, 1.76 mmol, 1.1 equiv, 60% in paraffin) was then added and mixture stirred for 15 min at 0 °C. After the dropwise addition of iodomethane (227 mg, 1.60 mmol, 1.0 equiv) the mixture was allowed to warm to ambient temperature. After 1.5 h, EtOAc was added to the solution and the organic layer was washed with brine (3 × 25 mL), dried over $Na_2SO_4$ and the solvent was removed under reduced pressure. The crude was purified using a silica column with a solvent gradient 5:95-20:80 (EtOAc /hexane) to obtain 186 mg of colourless oil (57% yield).[3] Isolated yield: 0.186 g (57%, 1.6 mmol), colorless oil; Rf: 0.25 (1/9, EtOAc/hexane); $^1$H NMR (700 MHz, $CDCl_3$) δ/ppm 7.56–7.51 (m, 2H), 7.41 (tt, *J* = 6.7, 0.9 Hz, 2H), 7.39–7.35 (m, 1H), 4.29–4.19 (m, 2H), 1.95 (s, 3H), 1.25 (t, *J* = 7.1, 3H); $^{13}$C NMR (176 MHz, $CDCl_3$) δ/ppm 167.9, 135.8, 129.1, 128.8, 125.7, 119.5, 63.2, 48.3, 24.9, 13.8; IR (neat) ν = 2986.2 (w), 2941.9 (w), 2363.5 (w), 2245.8 (w), 1962.1 (w), 1740.7 (s), 1599.6 (w), 1989.9 (w), 1493.6 (m), 1447.0 (s), 1379.7 (m), 1367.0 (w), 1297.1 (w), 1235.3 (s), 1149.8 (m), 1099.8 (s), 1077.4 (m), 1012.9 (s), 933,7 (w), 914.1 (w), 893.5 (w), 854.8 (m), 810.3 (w), 767.0 (s), 728.6 (s), 695.7 (s), 645.5 (m), 618.3 (w), 610.0 (w) $cm^{-1}$; LC-MS (MeCN), Rt. 3.05 min, m/z = 203.9 $[M+H]^+$. HR-MS ($^+$ESI-TOF) calculated for $C_{12}H_{14}NO_2S$ 204.1025, found 204.1027 (Δ = 1.0 ppm).

**Ethyl 2-cyano-3-methylbutanoate (47):**

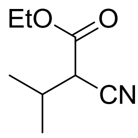


Chemical Formula: $C_8H_{13}NO_2$
Exact Mass: 155.0946

A solution of ethyl 2-cyanocetate (1.996 g, 17.64 mmol) in dry THF (3 mL) was slowly added to a suspension of sodium hydride (0.235 g, 5.88 mmol, 60% in paraffin) and THF (5 mL) under nitrogen atmosphere at 0 °C. After 15 min, a solution of 2-iodopropane (1.00 g, 5.88 mmol) in THF (3 mL) was added dropwise and after stirring the suspension for 15 min at 0 °C, the reaction mixture was warmed to room temperature and stirred for 8 h. The reaction was then quenched with 1 M HCl (10 mL) and extracted using EtOAc (2 × 25 mL). The organic layer was dried over $Na_2SO_4$ and the solvent was evaporated under reduced pressure to give the crude as an orange oil. The crude was purified on a silica column (1:4, EtOAc/hexanes) to give the desired product. Isolated yield: 0.557 g (61%, 5.88 mmol scale), colourless oil; $^1$H NMR (400 MHz, $CDCl_3$) δ/ppm 4.27 (qd, *J* = 7.1, 0.8 Hz, 2H), 3.39 (d, *J* = 5.3 Hz, 1H), 2.42 (pd, *J* = 6.8, 5.3 Hz, 1H), 1.32 (t, *J* = 7.1 Hz, 3H), 1.14 (dd, *J* = 12.3, 6.8 Hz, 6H); $^{13}$C NMR (101 MHz, $CDCl_3$) δ/ppm 166.1, 115.6, 62.8, 45.5, 30.1, 20.8, 19.0, 14.2; IR (neat) ν = 2972 (m), 2936 (w), 2878 (w), 1743 (s), 1467 (w), 1394 (w), 1370 (w), 1304 (w), 1253 (m), 1192 (m), 1133 (w), 1030 (m), 856 (w) $cm^{-1}$; LC-MS (MeCN), Rt. 2.55 min, m/z = 156.1 $[M+H]^+$. HR-MS ($^+$ESI-TOF) calculated for $C_8H_{14}NO_2$ 155.1021, found 156.1025 (Δ = 2.6 ppm).
